# Prediction of new associations between ncRNAs and diseases exploiting multi-type hierarchical clustering

**DOI:** 10.1186/s12859-020-3392-2

**Published:** 2020-02-24

**Authors:** Emanuele Pio Barracchia, Gianvito Pio, Domenica D’Elia, Michelangelo Ceci

**Affiliations:** 1 University of Bari Aldo Moro - Department of Computer Science, Via Orabona, 4, Bari, 70125 Italy; 2 Big Data Laboratory, National Interuniversity Consortium for Informatics (CINI), Rome, 00185 Italy; 3 CNR, Institute for Biomedical Technologies, Bari, 70126 Italy; 4 Department of Knowledge Technologies, Jožef Stefan Institute, Jamova 39, Ljubljana, 1000 Slovenia

**Keywords:** Non-coding RNA (ncRNAs), Diseases, Cancer, Heterogeneous network, Clustering, Link prediction

## Abstract

**Background:**

The study of functional associations between ncRNAs and human diseases is a pivotal task of modern research to develop new and more effective therapeutic approaches. Nevertheless, it is not a trivial task since it involves entities of different types, such as microRNAs, lncRNAs or target genes whose expression also depends on endogenous or exogenous factors. Such a complexity can be faced by representing the involved biological entities and their relationships as a network and by exploiting network-based computational approaches able to identify new associations. However, existing methods are limited to homogeneous networks (i.e., consisting of only one type of objects and relationships) or can exploit only a small subset of the features of biological entities, such as the presence of a particular binding domain, enzymatic properties or their involvement in specific diseases.

**Results:**

To overcome the limitations of existing approaches, we propose the system LP-HCLUS, which exploits a multi-type hierarchical clustering method to predict possibly unknown ncRNA-disease relationships. In particular, LP-HCLUS analyzes heterogeneous networks consisting of several types of objects and relationships, each possibly described by a set of features, and extracts multi-type clusters that are subsequently exploited to predict new ncRNA-disease associations. The extracted clusters are overlapping, hierarchically organized, involve entities of different types, and allow LP-HCLUS to catch multiple roles of ncRNAs in diseases at different levels of granularity. Our experimental evaluation, performed on heterogeneous attributed networks consisting of microRNAs, lncRNAs, diseases, genes and their known relationships, shows that LP-HCLUS is able to obtain better results with respect to existing approaches. The biological relevance of the obtained results was evaluated according to both quantitative (i.e., TPR@k, Areas Under the TPR@k, ROC and Precision-Recall curves) and qualitative (i.e., according to the consultation of the existing literature) criteria.

**Conclusions:**

The obtained results prove the utility of LP-HCLUS to conduct robust predictive studies on the biological role of ncRNAs in human diseases. The produced predictions can therefore be reliably considered as new, previously unknown, relationships among ncRNAs and diseases.

## Background

High-throughput sequencing technologies, together with recent, more efficient computational approaches have been fundamental for the rapid advances in functional genomics. Among the most relevant results, there is the discovery of thousands of non-coding RNAs (ncRNAs) with a regulatory function on gene expression [1]. In parallel, the number of studies reporting the involvement of ncRNAs in the development of many different human diseases has grown exponentially [2]. The first type of ncRNAs that has been discovered and largely studied is that of microRNAs (miRNAs), classified as small non-coding RNAs in contrast with the other main category represented by long non-coding RNAs (lncRNAs), that are ncRNAs longer than 200nt [3, 4].

Long non-coding RNAs (lncRNAs) and microRNAs (miRNAs) [5] are among the largest and heterogeneous groups of regulators of major cellular processes. However, lncRNAs, differently from miRNAs which primarily act as post-transcriptional regulators, have a plethora of regulatory functions [6]. They are involved in chromatin remodeling and epigenetic modifications, and organize functionally different nuclear sub-compartments with an impact on the nuclear architecture [7]. LncRNAs are also involved in the regulation of the expression of transcripts at cytoplasmic level by another series of interactions/functions that interfere with the efficiency of translation of transcripts in their protein products. In particular, they can directly interfere with miRNAs functions acting as miRNA sponges [8]. Nevertheless, the number of lncRNAs for which the functional and molecular mechanisms are completely elucidated is still quite poor. This is due to two main reasons: their recent discovery as master regulators with respect to miRNAs, and some particular features, such as the low cross-species conservation, the low expression levels and the high tissue specificity that make their characterization or any type of generalization still very difficult [9]. Therefore, assessing the role and the molecular mechanisms underlying the involvement of lncRNAs in human diseases is not a trivial task, and experimental investigations are still too much expensive for being carried out without any computational pre-analysis.

In the last few years, there have been several attempts to computationally predict the relationships among biological entities, such as genes, miRNAs, lncRNAs, diseases, etc. [10–19]. Such methods are mainly based on a network representation of the entities under study and on the identification of new links among nodes in the network. However, most of the existing approaches are able to work only on homogeneous networks (where nodes and links are of one single type) [20], are strongly limited by the number of different node types or are constrained by a pre-defined network structure. To overcome these limitations we propose the method LP-HCLUS (Link Prediction through Hierarchical CLUStering), which can discover previously unknown ncRNA-disease relationships working on heterogeneous attributed networks (that is, networks composed of different biological entities related by different types of relationships) with arbitrary structure. This ability allows LP-HCLUS to investigate how different types of entities interact with each other, possibly leading to increased prediction accuracy. LP-HCLUS exploits a combined approach based on hierarchical, multi-type clustering and link prediction. As we will describe in detail in the next section, a multi-type cluster is actually a heterogeneous sub-network. Therefore, the adoption of a clustering-based approach allows LP-HCLUS to base its predictions on relevant, highly-cohesive heterogeneous sub-networks. Moreover, the hierarchical organization of clusters allows it to perform predictions at different levels of granularity, taking into account either local/specific or global/general relationships.

Methodologically, LP-HCLUS estimates an initial score for each possible relationship involving entities belonging to the types of interest (in our case, ncRNAs and diseases), by exploiting the whole network. Such scores are then used to identify a hierarchy of overlapping multi-type clusters, i.e., groups of objects of different types. Finally, the identified clusters are exploited to predict new relationships, each of which is associated with a score representing its degree of certainty. Therefore, according to the classification provided in [21] (see Additional file 1), LP-HCLUS simultaneously falls in two categories: *i) algorithmic* methods, since it strongly relies on a clustering approach to predict new relationships and to associate them with a score in [0,1], and *ii) similarity-based* approaches, since the first phase (see “Estimation of the strength of the relationship between ncRNAs and diseases” section) exploits the computation of similarities between target nodes, taking into account the paths in the network and the attributes of the nodes.

The rest of the paper is organized as follows: in the next section, we describe our method for the identification of new ncRNA-disease relationships; in “Results” section we describe our experimental evaluation and in “Discussion” section we discuss the obtained results, including a qualitative analysis of the obtained predictions; finally, we conclude the paper and outline some future work. Moreover, in Additional file 1, we discuss the works related to the present paper; in Additional file 2 we report an analysis of the computational complexity of the proposed method; finally, in Additional files 3, 4 and 5 we report some detailed results obtained during the experiments.

## Methods

The algorithmic approach followed by LP-HCLUS mainly relies on the predictive clustering framework [22–24]. The motivation behind the adoption of such a framework comes from its recognized ability of handling data affected by different forms of autocorrelation, i.e., when close objects (spatially, temporally, or in a network as in this work) appear to be more similar than distant objects. This peculiarity allows LP-HCLUS to catch multiple dependencies among the involved entities, which can represent relevant cooperative/interfering activities.

Specifically, LP-HCLUS identifies hierarchically organized, possibly overlapping multi-type clusters from a heterogeneous network and exploits them for predictive purposes, i.e., to predict the existence of previously unknown links. The extraction of a hierarchical structure, rather than a flat structure, allows the biologists to focus on either more general or more specific interaction activities. Finally, the possible overlaps among the identified clusters allow LP-HCLUS to consider multiple roles of the same disease or ncRNA, which may be involved in multiple interaction networks.

It is noteworthy that, even if the analyzed network may consist of an arbitrary number of types of nodes and edges, the prediction of new associations will focus on edges involving ncRNAs and diseases, called *target* types. On the contrary, node types that are only used during the analysis will be called *task-relevant* node types.

Intuitively, the approach followed by LP-HCLUS consists of three main steps:

1. estimation of the strength of relationships for all the possible pairs of ncRNAs and diseases, according to the paths connecting such nodes in the network and to the features of nodes involved in such paths;
2. construction of a hierarchy of overlapping multi-type clusters, on the basis of the strength of relationships computed in the previous step;
3. identification of predictive functions to predict new ncRNA-disease relationships on the basis of the clusters identified at different levels of the hierarchy.

It is noteworthy that the clustering step could be directly applied on the set of known interactions, without performing the first step. However, such an approach would lead to discard several potential indirect relationships that can be caught only through a deep analysis of the network, which is indeed the main purpose of the first step. A naïve solution for the prediction task would be the use of the output of the first step as the final score, ignoring steps 2 and 3. However, this would lead to disregard a more abstract perspective of the interactions which, instead, can be caught by the clustering-based approach. Another effect would be to disregard the network homophily phenomenon and not to catch possible relationships between ncRNAs and between diseases based on the nodes they are connected with. On the contrary, the exploitation of such relationships is in line with the *guilt-by-association (GBA)* principle, which states that entities with similar functions tend to share interactions with other entities. This principle has been recently applied to and investigated for ncRNAs [25].

Each step will be described in details in the next subsections, while in the following we formally define the heterogeneous attributed network, that is analyzed by LP-HCLUS, as well as the solved task.

### **Definition 1**

**Fig. 1:**
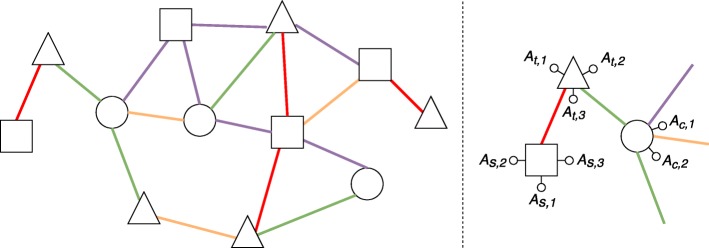
An example of a heterogeneous attributed network. On the left, a general overview of the network, where shapes represent different node types and colors represent different edge types. On the right, a zoom on a small portion of the network, where we can observe node attributes associated with squares (*A*_*s*,∗_), triangles (*A*_*t*,∗_) and circles (*A*_*c*,∗_)
- each node type $T_{v} \in \mathcal {T}$ defines a subset of nodes in the network, that is *V*_*v*_⊆*V*;
- each node type $T_{v} \in \mathcal {T}$ is associated with a set of attributes $\mathcal {A}_{v} = \{A_{v,1}, A_{v,2},\ldots, A_{v,m_{v}}\}$, i.e., all the nodes of a given type *T*_*v*_ are described according to the attributes $\mathcal {A}_{v}$;
- $\mathcal {R}$ is the set of all the possible edge types;
- each edge type $R_{l} \in \mathcal {R}$ defines a subset of edges *E*_*l*_⊆*E*.

(Heterogeneous attributed network) A heterogeneous attributed network is a network *G*=(*V*,*E*), where *V* denotes the set of nodes and *E* denotes the set of edges, and both nodes and edges can be of different types (see Fig. 1). Moreover:

- $\mathcal {T} = \mathcal {T}_{t} \cup \mathcal {T}_{tr}$ is the set of node types, where $\mathcal {T}_{t}$ is the set of target types and $\mathcal {T}_{tr}$ is the set of task-relevant types;

### **Definition 2**

(Overlapping Multi-type cluster) Given a heterogeneous attributed network *G*=(*V*,*E*), an overlapping multi-type cluster is defined as *G*^′^=(*V*^′^,*E*^′^), where:

- *V*^′^⊆*V*;
- ∀*v*^′^∈*V*^′^,*v*^′^ is a node of a target type;
- ∀*v*^′^∈*V*^′^,*v*^′^ may also belong to other clusters besides *G*^′^;
- $E' \subseteq (E \cup \hat {E})$ is a set of relationships among the nodes in *V*^′^, belonging either to the set of known relationships *E* or to a set of *extracted* relationships $\hat {E}$, which are identified by the clustering method.

The details about the strategy adopted to identify $\hat {E}$ will be discussed in “Estimation of the strength of the relationship between ncRNAs and diseases” section.

### **Definition 3**

**Fig. 2:**
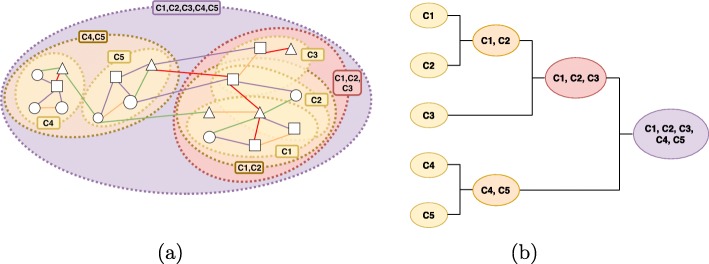
A hierarchy of overlapping multi-type clusters: **a** emphasizes the overlapping among multi-type clusters; **b** shows their hierarchical organization

(Hierarchical multi-type clustering) A hierarchy of multi-type clusters is defined as a list of hierarchy levels [*L*_1_,*L*_2_,…,*L*_*k*_], where each *L*_*i*_ consists of a set of overlapping multi-type clusters. For each level *L*_*i*_,*i*=2,3,..…*k*, we have that ∀ *G*^′^∈*L*_*i*_ ∃ *G*^″^∈*L*_*i*−1_, such that *G*^″^ is a subnetwork of *G*^′^ (see Fig. 2).

On the basis of these definitions, we formally define the task considered in this work.

### **Definition 4**

(Predictive hierarchical clustering for link prediction) Given a heterogeneous attributed network *G*=(*V*,*E*) and the set of target types $\mathcal {T}_{t}$, the goal is to find:

- A hierarchy of overlapping multi-type clusters [*L*_1_,*L*_2_,…,*L*_*k*_].
- A function $\psi ^{(w)}:V_{i_{1}} \times V_{i_{2}} \rightarrow [0,1]$ for each hierarchical level *L*_*w*_ (*w*∈1,2,...,*k*), where nodes in $V_{i_{1}}$ are of type $T_{i_{1}} \in \mathcal {T}_{t}$ and nodes in $V_{i_{2}}$ are of type $T_{i_{2}} \in \mathcal {T}_{t}$. Intuitively, each function *ψ*^(*w*)^ maps each possible pair of nodes (of types $T_{i_{1}}$ and $T_{i_{2}}$, respectively) to a score that represents the degree of certainty of their relationship.

The learning setting considered in this paper is *transductive*. In particular, only the links involving nodes already known and exploited during the training phase are considered for link prediction. In other terms, we do not learn a model from a network and apply this model to a completely different network (classical inductive learning setting).

**Fig. 3:**
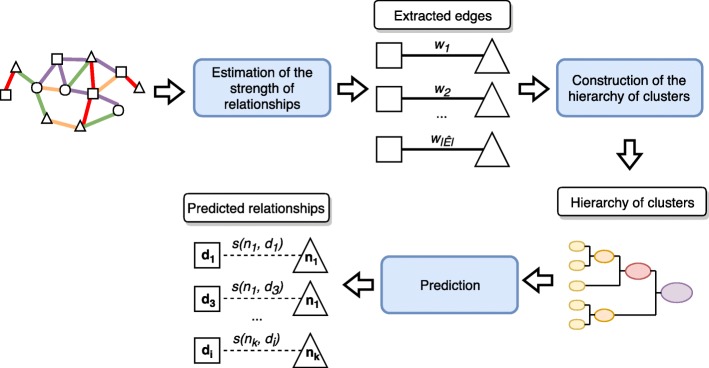
Workflow of the method LP-HCLUS

**Fig. 4:**
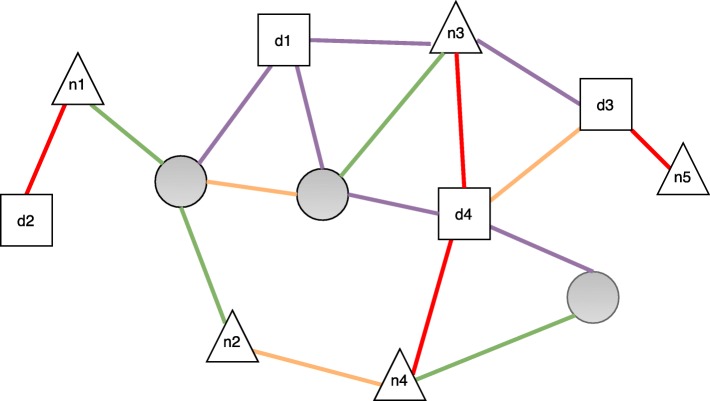
An example of a ncRNA-disease heterogeneous network. In this example, ncRNAs are represented as triangles, while diseases are represented as squares. Other (task-relevant) nodes (e.g., target genes, proteins, etc) are represented as gray circles

The method proposed in this paper (see Fig. 3 for the general workflow) aims at solving the task formalized in Definition 4, by considering ncRNAs and diseases as target types (Fig. 4). Hence, we determine two distinct set of nodes denoted by *T*_*n*_ and *T*_*d*_, representing the set of ncRNAs and the set of diseases, respectively.

### Estimation of the strength of the relationship between ncRNAs and diseases

**Fig. 5:**
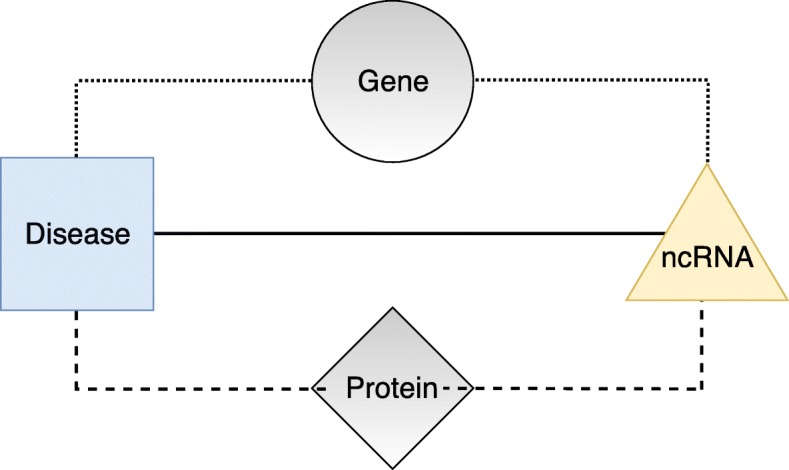
Diagram showing three different meta-paths between a disease and a ncRNA. The first meta-path connects diseases and ncRNAs via genes, the second connects diseases and ncRNAs directly and the third connects diseases and ncRNAs via proteins

In the first phase, we estimate the strength of the relationship among all the possible ncRNA-disease pairs in the network *G*. In particular, we aim to compute a score *s*(*n*_*i*_,*d*_*j*_) for each possible pair *n*_*i*_,*d*_*j*_, by exploiting the concept of *meta-path*. According to [26], a *meta-path* is a set of sequences of nodes which follow the same sequence of edge types, and can be used to fruitfully represent conceptual (possibly indirect) relationships between two entities in a heterogeneous network (see Fig. 5). Given the ncRNA *n*_*i*_ and the disease *d*_*j*_, for each meta-path *P*, we compute a score *pathscore*(*P*,*n*_*i*_,*d*_*j*_), which represents the strength of their relationship on the basis of the meta-path *P*.

In order to combine multiple contributions provided by different meta-paths, we adopt a strategy that follows the classical formulation of fuzzy sets [27]. In particular, a relationship between a ncRNA *n*_*i*_ and a disease *d*_*j*_ can be considered “certain” if there is at least one meta-path which confirms its certainty. Therefore, by assimilating the score associated with an interaction to its degree of certainty, we compute *s*(*n*_*i*_,*d*_*j*_) as the maximum value observed over all the possible meta-paths between *n*_*i*_ and *d*_*j*_. Formally:

1 $$ s(n_{i},d_{j})= \max_{P \in metapaths(n_{i},d_{j})} pathscore(P,n_{i}, d_{j})  $$

where *metapaths*(*n*_*i*_,*d*_*j*_) is the set of meta-paths connecting *n*_*i*_ and *d*_*j*_, and *pathscore*(*P*,*n*_*i*_,*d*_*j*_) is the degree of certainty of the relationship between *n*_*i*_ and *d*_*j*_ according to the meta-path *P*.

As introduced before, each meta-path *P* represents a finite set of sequences of nodes, where:

- the *i*-th node of each sequence in the metapath *P* is of the same type;
- the first node is a ncRNA and the last node is a disease;
- if two nodes are consecutive in the sequence, then there is an edge between them in *E*.

According to this definition, if there is a path *P* directly connecting a ncRNA *n*_*i*_ to a disease *d*_*j*_, then *pathscore*(*P*,*n*_*i*_,*d*_*j*_)=1, therefore *s*(*n*_*i*_,*d*_*j*_)=1.

Otherwise, when there is no direct connection between *n*_*i*_ and *d*_*j*_,*pathscore*(*P*,*n*_*i*_,*d*_*j*_) is computed as the maximum similarity between the sequences that start with *n*_*i*_ and those that end with *d*_*j*_. Formally:

2 $$\begin{array}{*{20}l} &pathscore (P, n_{i}, d_{j})= \\ & \quad\qquad \max_{\substack{seq', seq^{\prime\prime} \in P,\\ seq'.first=n_{i}, seq^{\prime\prime}.last=d_{j}}} similarity (seq', seq^{\prime\prime})  \end{array} $$

The intuition behind this formula is that if *n*_*i*_ and *d*_*j*_ are not directly connected, their score represents the similarity of the nodes and edges they are connected to. In other words, this is a way to analyze the similarity between the neighborhood of *n*_*i*_ and the neighborhood of *d*_*j*_ in terms of the (similarity of the) paths they are involved in.

It is noteworthy that, in order to make the neighbors comparable, we exploit the concept of meta-path, which includes sequences that involve the same types of nodes. In fact, in Formula (2), the similarity between two sequences *seq*^′^ and *seq*^′′^ is computed as follows:

3 $$ similarity(seq',seq^{\prime\prime}) = \frac{\sum_{x \in A^{(P)}} s_{x}(seq',seq^{\prime\prime})}{|A^{(P)}|}   $$

where:

- *A*^(*P*)^ is the set of attributes of the nodes involved in the path *P*;
- *s*_*x*_(*seq*^′^,*seq*^′′^) is the similarity between *val*_*x*_(*seq*^′^), that is the value of the attribute *x* in the sequence *seq*^′^, and *val*_*x*_(*seq*^′′^), that is the value of the attribute *x* in the sequence *seq*^′′^.

Following [28], we compute *s*_*x*_(*seq*^′^,*seq*^′′^) as follows:

- if *x* is numeric, then $s_{x}(seq',seq^{\prime \prime }) = 1 - \frac {|val_{x}(seq') - val_{x}(seq^{\prime \prime })|}{max_{x} -min_{x}}$, where *min*_*x*_ (resp. *max*_*x*_) is the minimum (resp. maximum) value, for the attribute *x*;
- if *x* is not a numeric attribute, then *s*_*x*_(*seq*^′^,*seq*^′′^)=1 if *val*_*x*_(*seq*^′^)=*val*_*x*_(*seq*^′′^), 0 otherwise.

**Fig. 6:**
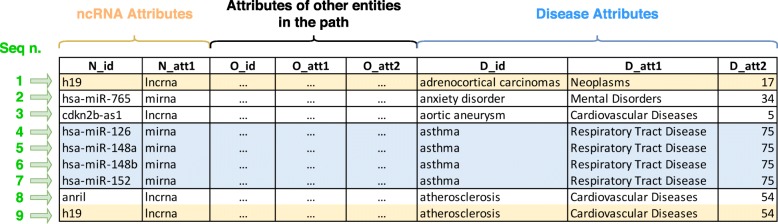
Analysis of sequences between the ncRNA “h19” and the disease “asthma” according to a meta-path. Sequences emphasized in yellow (1 and 9) are those starting with “h19”, while sequences emphasized in blue (4, 5, 6 and 7) are those ending with “asthma”. White rows, although belonging to P, are not considered during the computation of the similarity in this specific example, since they do not involve “h19” or “asthma”

An example of the computation of the similarity among sequences is reported in Fig. 6. In this example, we compute the score between the ncRNA *h19* and the disease *asthma*. First, we identify the sequences starting with *h19* (i.e., 1 and 9, emphasized in yellow) and those ending with *asthma* (i.e., 4, 5, 6 and 7, emphasized in blue). Then we pairwisely compute the similarity between sequences belonging to the two sets and select the maximum value, according to Eq. 2. The similarity between two sequences is computed according to Eq. 3.

In this solution there could be some node types that are not involved in any meta-path. In order to exploit the information conveyed by these nodes, we add an aggregation of their attribute values (the *arithmetic mean* for numerical attributes, the *mode* for non-numerical attributes) to the nodes that are connected to them and that appear in at least one meta-path. Such an aggregation is performed up to a predefined *depth* of analysis in the network. In this way, we fully exploit the network autocorrelation phenomena.

### Construction of a hierarchy of overlapping multi-type clusters

**Fig. 7:**
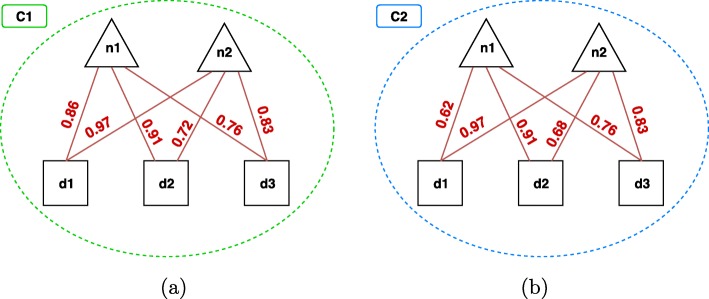
Biclique constraint on two multi-type clusters **a** An example of multi-type cluster which satisfies the biclique constraint with *β*=0.7 (i.e., all the relationships have a score ≥0.7). **b** An example that does not satisfy such a constraint. It is noteworthy that, with *β*=0.6, also (**b**) would satisfy the biclique constraint
ii) **Initialization**, which builds the initial set of clusters in the form of bicliques, each consisting of a ncRNA-disease pair in {(*n*_*i*_,*d*_*j*_)|*s*(*n*_*i*_,*d*_*j*_)≥*β*}.
iii) **Merging**, which iteratively merges two clusters *C*^′^ and *C*^″^ into a new cluster *C*^‴^. This step regards the initial set of clusters as a list sorted according to an ordering relation <_*c*_ that reflects the quality of the clusters. Each cluster *C*^′^ is then merged with the first cluster *C*^″^ in the list that would lead to a cluster *C*^‴^ which still satisfies the biclique constraint. This step is repeated until no additional clusters that satisfy the biclique constraint can be obtained.

Starting from the set of possible ncRNA-disease pairs, each associated with a score that represents its degree of certainty, we construct the first level of the hierarchy by identifying a set of overlapping multi-type clusters in the form of bicliques. That is, multi-type clusters where all the ncRNA-disease relationships have a score greater than (or equal to) a given threshold *β*∈[0,1] (see Fig. 7). More formally, in order to construct the first level of the hierarchy *L*_1_, we perform the following steps:

i) **Filtering**, which keeps only the ncRNA-disease pairs with a score greater than (or equal to) *β*. The result of this step is the subset {(*n*_*i*_,*d*_*j*_)|*s*(*n*_*i*_,*d*_*j*_)≥*β*}.

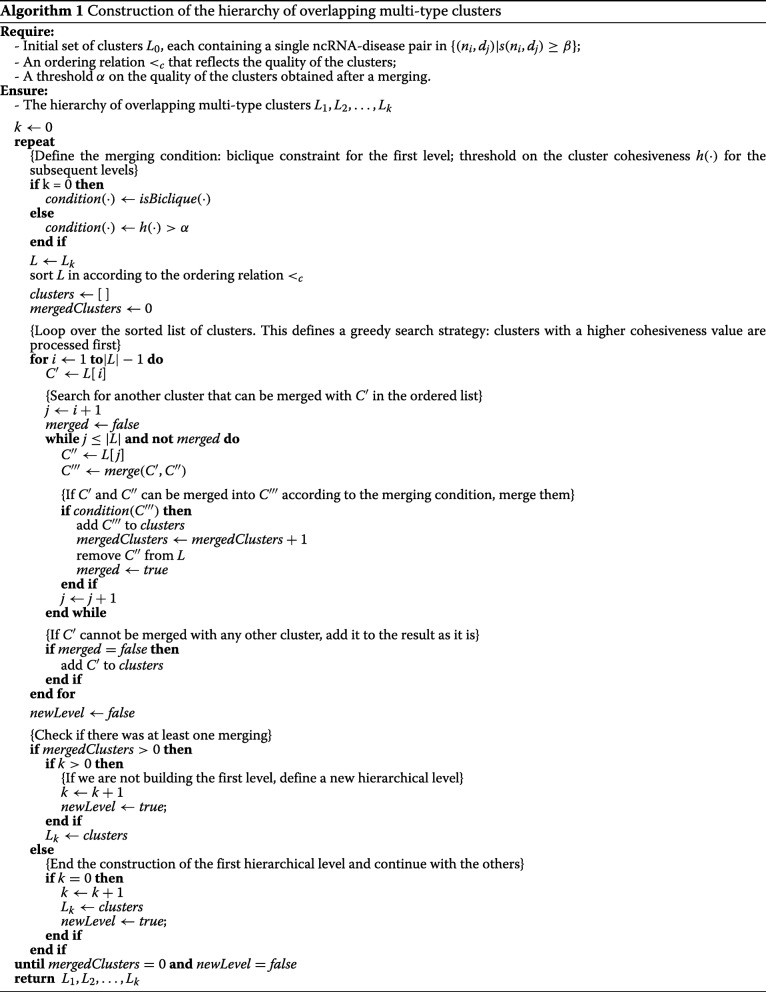


The ordering relation <_*c*_ exploited by the merging step implicitly defines a greedy search strategy that guides the order in which pairs of clusters are analyzed and possibly merged. <_*c*_ is based on the cluster cohesiveness *h*(*c*), which corresponds to the average score of the interactions in the cluster. Formally:

4 $$ h(C) = \frac{1}{|pairs(C)|} \cdot {\sum_{(n_{i},d_{j}) \in pairs(C)} s(n_{i},d_{j})}   $$

where *pairs*(*C*) is the set of all the possible ncRNA-disease pairs that can be constructed from the set of ncRNAs and diseases in the cluster. Numerically, |*pairs*(*C*)|=|{*n*_*i*_|*n*_*i*_∈*C*∧*n*_*i*_∈*T*_*n*_}|·|{*d*_*j*_|*d*_*j*_∈*C*∧*d*_*j*_∈*T*_*d*_}|.

Accordingly, if *C*^′^ and *C*^′′^ are two different clusters, the ordering relation <_*c*_ is defined as follows:

5 $$ C' <_{c} C^{\prime\prime} \iff h(C') > h(C^{\prime\prime})   $$

The approach adopted to build the other hierarchical levels is similar to the merging step performed to obtain *L*_1_. The main difference is that, in this case, we do not obtain bicliques, but generic multi-type clusters, i.e., the score associated with each interaction does not need to satisfy the threshold *β*. Since the biclique constraint is removed, we need another stopping criterion for the iterative merging procedure. Coherently with approaches used in hierarchical co-clustering and following [29], we adopt a user-defined threshold *α* on the cohesiveness of the obtained clusters. In particular, two clusters *C*^′^ and *C*^′′^ can be merged into a new cluster *C*^′′′^ if *h*(*C*^′′′^)>*α*, where *h*(*C*^′′′^) is the cluster cohesiveness defined in Eq. 4. This means that *α* defines the minimum cluster cohesiveness that must be satisfied by a cluster obtained after a merging: small values of *α* lead to increase the number of merging operations and, therefore, to a relatively small number of final clusters containing a large number of nodes.

For every iteration of the merging procedure, a new hierarchical level is generated. The iterative process stops when it is not possible to merge more clusters with a minimum level of cohesiveness *α*. The output of such a process is a hierarchy of overlapping multi-type clusters {*L*_1_,*L*_2_,…,*L*_*k*_} (see Definition 3).

A pseudocode description of the proposed algorithm for the construction of the hierarchy of clusters is reported in Algorithm 1.

### Prediction of new ncRNA-disease relationships

In the last phase, we exploit each level of the identified hierarchy of multi-type clusters as a prediction model. In particular, we compute, for each ncRNA-disease pair, a score representing its degree of certainty on the basis of the multi-type clusters containing it. Formally, let $C_{ij}^{w}$ be a cluster identified in the *w*-th hierarchical level in which the ncRNA *n*_*i*_ and the disease *d*_*j*_ appear. We compute the degree of certainty of the relationship between *n*_*i*_ and *d*_*j*_ as:

6 $$ \psi^{(w)}(n_{i}, d_{j}) = h\left(C_{ij}^{w}\right),  $$

that is, we compute the degree of certainty of the new interaction as the average degree of certainty of the known relationships in the cluster. In some cases, the same interaction may appear in multiple clusters, since the proposed algorithm is able to identify overlapping clusters. In this case, $C_{ij}^{w}$ represents the list of multi-type clusters (i.e., $C^{w}_{ij} = [C_{1},C_{2}, \ldots, C_{m} ]$), ordered accordingly to relation <_*c*_ defined in Eq. 5, in which both *n*_*i*_ and *d*_*j*_ appear, on which we apply an aggregation function to obtain a single degree of certainty. In this work, we propose the adoption of four different aggregation functions:

- **Maximum**: $\psi ^{(w)}(n_{i}, d_{j}) = \max _{c \in C_{ij}^{w}} h(c)$
- **Minimum**: $\psi ^{(w)}(n_{i}, d_{j}) = \min _{c \in C_{ij}^{w}} h(c)$
- **Average**: $ \psi ^{(w)}(n_{i}, d_{j}) = \frac {1}{|C_{ij}^{w}|} \cdot {\sum _{c \in C_{ij}^{w}} h(c)} $
- **Evidence Combination**: *ψ*^(*w*)^(*n*_*i*_,*d*_*j*_)=*ec*(*C*_*m*_), where:
  7 $$ {}ec(C_{m}) \!= \! \left\{ \!\!\begin{array}{ll} h(C_{1}) & \ \text{if}\ C_{m} \!= \! C_{1}\\ ec(C_{m-1}) + [1-ec(C_{m-1})] \cdot h(C_{m}) & \ \text{otherwise} \end{array}\right.   $$

It is noteworthy that the Evidence Combination function, already exploited in the literature in the context of expert systems [30], generally rewards the relationships appearing in multiple high cohesive clusters.

**Fig. 8:**
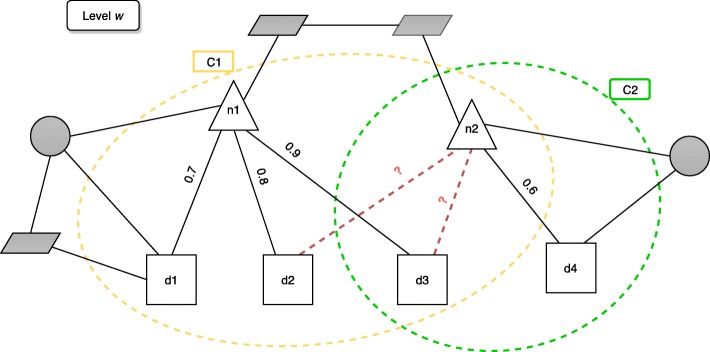
Example of the prediction step. Two clusters identified at a given hierarchical level *w*. Triangles represent ncRNAs, squares represent diseases and the grey shapes are other type nodes. The clusters suggest two new possible relationships between *n*_2_ and *d*_2_ and between *n*_2_ and *d*_3_

In the following, we report an example of this prediction step, with the help of Fig. 8. In this example, we have two overlapping multi-type clusters *C*_1_ and *C*_2_, identified at the *w*-th hierarchical level, that suggest two new potential relationships (dashed lines in the figure), i.e. the pair *n*_2_,*d*_2_ and the pair *n*_2_,*d*_3_.

The first relationship only appears in *C*_1_, therefore its degree of certainty is computed according to the cohesiveness of *C*_1_ (see Eq. 4):

8 $$ {}\psi^{(w)}(n_{2}, d_{2}) = h(C_{1}) = \frac{1}{2 \cdot 3}(0.7 + 0.8 + 0.9) = 0.4.  $$

On the contrary, the second relationship is suggested by both *C*_1_ and *C*_2_, i.e., it appears in their overlapped area. Therefore, we aggregate the cohesiveness of *C*_1_ and *C*_2_ according to one of the functions we described before. In particular, since *h*(*C*_1_)=0.4 and $h(C_{2}) = \frac {1}{1 \cdot 2} \cdot 0.6 = 0.3$, we have:

- **Maximum**: $ \psi ^{(w)}(n_{2}, d_{3}) = \max _{c \in C_{ij}^{w}} h(c) = 0.4 $
- **Minimum**: $ \psi ^{(w)}(n_{2}, d_{3}) = \min _{c \in C_{ij}^{w}} h(c) = 0.3 $
- **Average**: $ \psi ^{(w)}(n_{2}, d_{3}) = \frac {1}{|C_{ij}^{w}|} \cdot {\sum _{c \in C_{ij}^{w}} h(c)} = \frac {1}{2} \cdot (0.4 + 0.3) = 0.35 $
- **Evidence Combination**: *ψ*^(*w*)^(*n*_2_,*d*_3_)=*h*(*C*_1_)+[1−*h*(*C*_1_)]·*h*(*C*_2_)=0.4+(1−0.4)·0.3=0.58

## Results

The proposed method was evaluated through several experiments. In this section, we present the main adopted resources, define the experimental setting, introduce the adopted evaluation measures and compare our system with the competitors from a quantitative viewpoint.

### Datasets

We performed experiments on two different heterogeneous networks involving ncRNAs and diseases. In the following, we report the details of each dataset, together with UML diagrams that represent their data and structure, i.e., nodes, links and attributes.

**Fig. 9:**
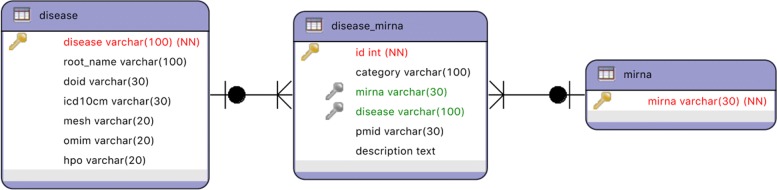
UML diagram of the dataset HMDD v3.0. The attributes in red are the identifiers of the nodes of a given type (i.e., the primary key in a relational database), while attributes in green refer to the identifier of nodes of other types (i.e., foreign keys in a relational database)

**HMDD v3** [31]. This dataset stores information about diseases, miRNAs and their known relationships. The network consists of 985 miRNAs, 675 diseases (characterized by 6 attributes) and 20,859 relationships between diseases and miRNAs (characterized by 3 attributes). A diagram of this dataset is depicted in Fig. 9, while the attributes are described in Table 1. The official link of the dataset is: http://www.cuilab.cn/hmdd. In this evaluation, we used two versions of the HMDD v3 dataset: the version released on June 28th, 2018 (v3.0) and the version released on March 27th, 2019 (v3.2). Both versions are available at the following link: http://www.di.uniba.it/~gianvitopio/systems/lphclus/.

**Table 1:** HMDD v3.0 dataset - Description of the attributes

Type	Feature	Description
*Disease*	disease	Disease name
	root_name	Category of the disease
	doid	Disease Ontology Identifiers
	icd10cm	ICD-10-CM Code
	mesh	Medical Subject Headings (MeSH) code
	omim	Online Mendelian Inheritance in Man (OMIM) code
	hpo	Human Phenotype Ontology (HPO) code
*Disease_miRNA*	id	ID of the relationship
	category	Category of the relationship
	mirna	miRNA involved in the association
	disease	Disease involved in the association
	pmid	PubMed ID of the publication reporting the association
	description	Description of the relationship
*miRNA*	mirna	miRNA name

**Integrated Dataset (ID)**. This dataset has been built by integrating multiple public datasets in a complex heterogeneous network. The source datasets are:

- lncRNA-disease relationships and lncRNA-gene interactions from [32] (June 2015)^1^
- miRNA-lncRNA interactions from [33] 2
- disease-gene relationships from DisGeNET v5 [34] 3
- miRNA-gene and miRNA-disease relationships from miR2Disease [35] 4

**Fig. 10:**
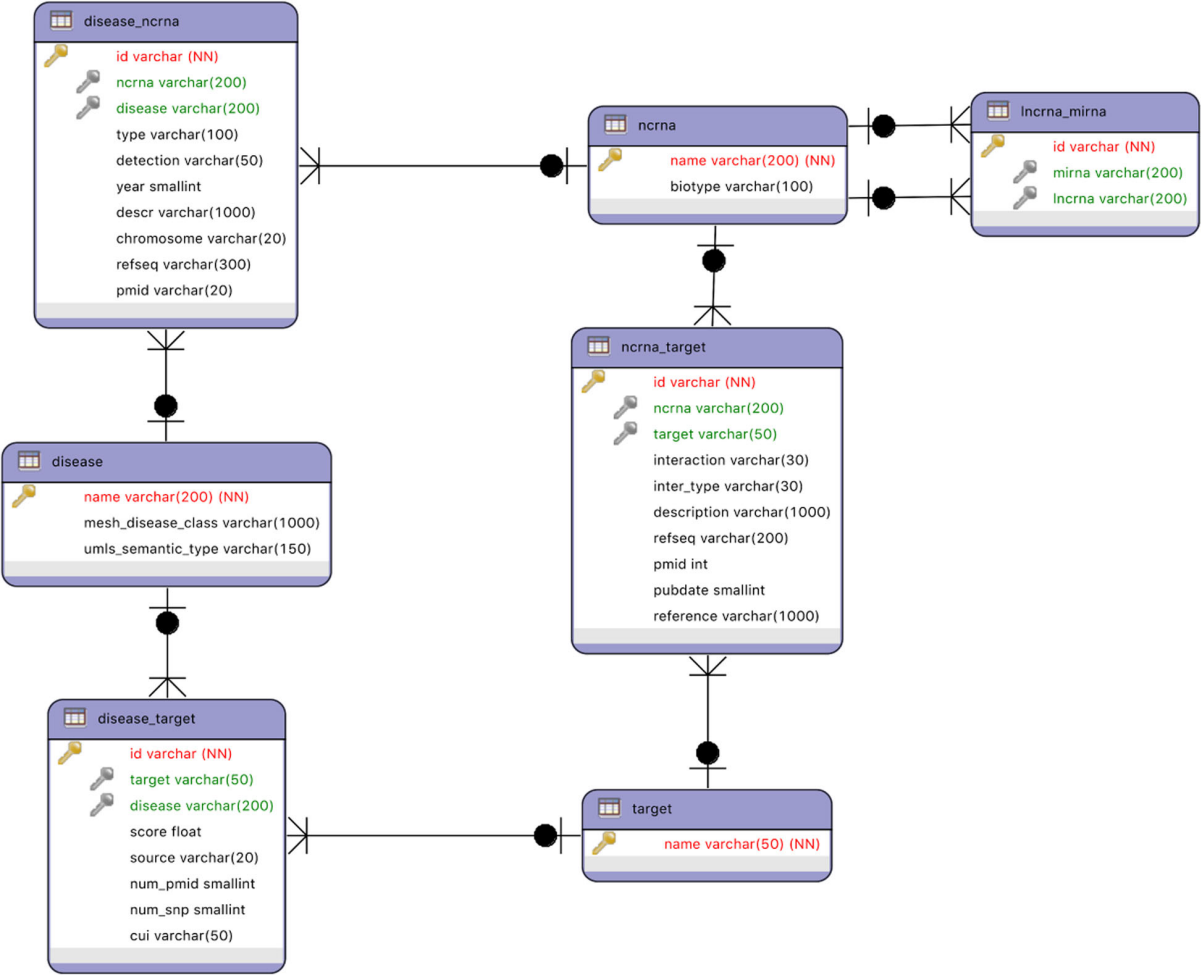
UML diagram of the Integrated Dataset (ID). The attributes in red are the identifiers of the nodes of a given type (i.e., the primary key in a relational database), while attributes in green refer to the identifier of nodes of other types (i.e., foreign keys in a relational database)

From these resources we only kept data related to *H. Sapiens*. The integration led to a network consisting of 1015 ncRNAs (either lncRNAs or miRNAs), 7049 diseases, 70 relationships between lncRNAs and miRNAs, 3830 relationships between diseases and ncRNAs, 90,242 target genes, 26,522 disease-target associations and 1055 ncRNA-target relationships. Most of the considered entities are also characterized by a variable number of attributes, as shown in Fig. 10 and in Table 2. The final dataset is available at the following link: http://www.di.uniba.it/~gianvitopio/systems/lphclus/.

**Table 2:** ID dataset - Description of the attributes

Type	Feature	Description
*Disease*	name	Disease name
	mesh_disease_class	Disease classification by Medical Subject Headings (MeSH)
	umls_semantic_type	Semantic type provided by the Unified Medical Language System
*Disease_ncRNA*	id	ID of the relationship
	ncrna	ncRNA involved in the association
	disease	Disease involved in the association
	type	Type of association
	detection	Method used to detect the relationship
	year	Year of the detection
	descr	Description of the association
	chromosome	Chromosome
	refseq	RefSeq identifier
	pmid	PubMed ID of the publication reporting the association
*Disease_target*	id	ID of the relationship
	target	Target gene involved in the association
	disease	Disease involved in the association
	score	DisGENET score for the Gene-Disease association
	source	Original source reporting the Gene-Disease association
	num_pmid	Total number of publications reporting the association
	num_snp	Total number of SNPs associated to the association
	cui	Concept Unique Identifier (CUI)
*lncRNA_miRNA*	id	ID of the relationship
	mirna	miRNA involved in the association
	lncrna	lncRNA involved in the association
*ncRNA*	name	ncRNA name
	biotype	Type of ncRNA. The value can be “lncrna” or “mirna”
*ncRNA_target*	id	ID of the relationship
	ncrna	ncRNA involved in the association
	target	Target genes involved in the association
	interaction	Elements involved in the associations (e.g. RNA-RNA, RNA-protein)
	inter_type	Type of interaction (e.g. Regulatory, Binding, etc.)
	description	Description of the interaction
	refseq	RefSeq identifier
	pmid	PubMed ID of the publication reporting the association
	pubdate	Date of first publication
	reference	Textual description of the association
*Target*	name	Name of target gene

### Experimental setting & competitors

LP-HCLUS has been run with different values of its input parameters, namely: *α*∈{0.1,0.2} (we remind that *α* is the minimum cohesiveness that a cluster must satisfy) and *β*∈{0.3,0.4} (we remind that *β* represents the minimum score that each ncRNA-disease pair must satisfy to be considered as existing), while *depth* has been set to 2 in order to consider only nodes that are relatively close to those involved in the meta-paths. We performed a comparative analysis with two competitor systems and a baseline approach that we describe in the following.

**HOCCLUS2** [29] is a biclustering algorithm that, similarly to LP-HCLUS, is able to identify a hierarchy of (possibly overlapping) heterogeneous clusters. HOCCLUS2 was initially developed to study miRNA-mRNA associations, therefore it is inherently limited to two target types. Moreover, besides miRNAs, mRNAs and their associations, it is not able to take into account other entities in the network and actually cannot predict new relationships. We adapted HOCCLUS2 in order to analyze ncRNA-disease relationships and to be able to predict new associations. In particular, we fed HOCCLUS2 with the dataset produced by the first step of LP-HCLUS (see “Estimation of the strength of the relationship between ncRNAs and diseases” section) and we performed the prediction according to the strategy we proposed for LP-HCLUS (see “Prediction of new ncRNA-disease relationships” section), considering all the aggregation functions proposed in this paper. We emphasize that, since both the initial analysis and the prediction step are performed by LP-HCLUS modules, the comparison with HOCCLUS2 allows us to evaluate the effectiveness of the proposed clustering approach. Since the HOCCLUS2 parameters have a similar meaning with respect to LP-HCLUS parameters, we evaluated its results with the same parameter setting, i.e., *α*∈{0.1,0.2} and *β*∈{0.3,0.4}.

**ncPred** [14] is a system which was specifically designed to predict new associations between ncRNAs and diseases. ncPred analyzes two matrices containing information about ncRNA-gene and gene-disease relationships. Therefore, we transformed the considered heterogeneous networks into matrices and fed ncPred with them. We again emphasize that ncPred is not able to catch information coming from other entities in the network of types different from ncRNAs and diseases, and that it is not able to exploit features associated to nodes and links in the network. We set ncPred parameter values to their default values.

**LP-HCLUS-NoLP**, which corresponds to our system LP-HCLUS, without the clustering and the link prediction steps. In particular, we consider the score obtained in the first phase of LP-HCLUS (see “Estimation of the strength of the relationship between ncRNAs and diseases” section) as the final score associated with each interaction. This approach allows us to evaluate the contribution provided by our link prediction approach based on multi-type clustering.

The evaluation was performed through a 10-fold cross-validation. It is noteworthy that the computation of classical measures, such as Precision and Recall, would require the presence of negative examples or some assumptions made on unknown examples. In our case, the datasets contain only positive examples, i.e., we have a set of validated relationships but we do not have negative examples of relationships (relationships whose non-existence has been proven).

Therefore, following the approach adopted in [13], we evaluated the results in terms of TruePositiveRate@*k*, where:

- an association is considered a True Positive (TP) if it is validated in the literature and it is in the first top *k* relationships predicted by the system;
- an association is considered a False Negative (or FN) if it is validated in the literature, but it is not in the first top *k* relationships predicted by the system.

Since the optimal value of *k* cannot be known in advance, we plot the obtained TPR@*k* by varying the value of *k* and compute the Area Under the TPR@*k* curve (AUTPR@*k*). For a thorough analysis on the most promising (i.e., top-ranked) interactions, we report all the results by varying the value of *k* within the interval [1,5000], obtained with the same configuration of the parameters *α* and *β* for HOCCLUS2 and LP-HCLUS. Moreover, we also report the results in terms of ROC and Precision-Recall curves, as well as the areas under the respective curves (AUROC and AUPR), by considering the unknown relationships as negative examples. We remark that AUROC and AUPR results can only be used for relative comparison and not as absolute evaluation measures because they are spoiled by the assumption made on unknown relationships.

In the paper we report the results obtained with the most promising configuration according to some preliminary experiments. The complete results, including those obtained in such preliminary experiments, can be downloaded at: http://www.di.uniba.it/~gianvitopio/systems/lphclus/.

### Results - HMDD v3 dataset

**Fig. 11:**
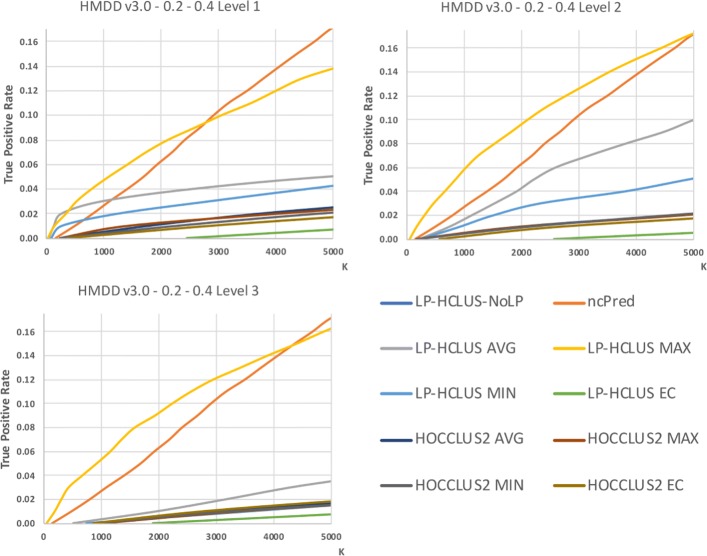
TPR@*k* results for the dataset HMDD v3.0, obtained with the best configuration (*α*=0.2,*β*=0.4) at different levels of the hierarchy

**Fig. 12:**
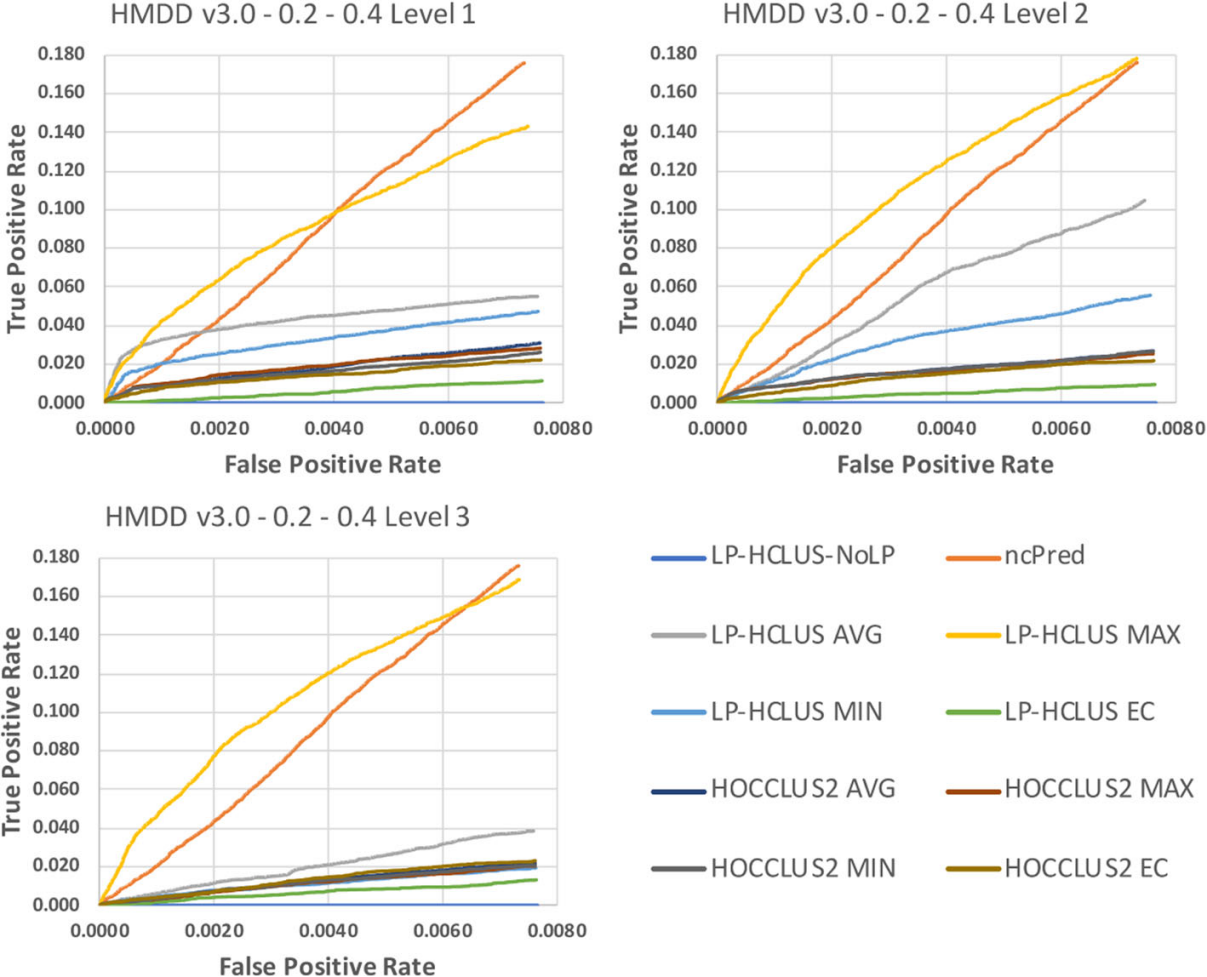
ROC curves for the dataset HMDD v3.0, obtained with the best configuration (*α*=0.2,*β*=0.4) at different levels of the hierarchy. These curves can only be used for relative comparison and not as absolute evaluation measures because they are spoiled by the assumption made on unknown relationships

**Fig. 13:**
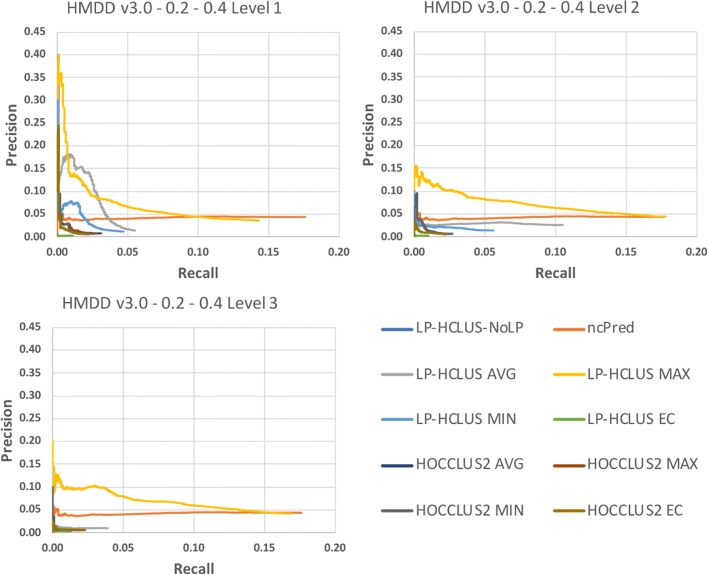
Precision-Recall curves for the dataset HMDD v3.0, obtained with the best configuration (*α*=0.2,*β*=0.4) at different levels of the hierarchy. These curves can only be used for relative comparison and not as absolute evaluation measures because they are spoiled by the assumption made on unknown relationships

In Figures 11, 12 and 13 we show the results obtained on the HMDD dataset in terms of TPR@k, ROC and Precision-Recall curves, while in Table 3, we report the AUTPR@*k*, AUROC and AUPR values. From Fig. 11, we can observe that the proposed method LP-HCLUS, with the combination strategy based on the maximum, is in general able to obtain the best performances. The competitor system ncPred obtains good results, but it outperforms LP-HCLUS_MAX only for high values of *k*, and only when focusing on the first level of the hierarchy. However, we stress the fact that it is highly preferable to achieve better performances on the left side of the curve, i.e., with low values of *k*, since it is the real portion of the ranking on which researchers will focus their analysis. In such a portion of the curve, LP-HCLUS_MAX dominates over all the competitors for all the hierarchical levels. It is noteworthy that some variants of LP-HCLUS (i.e., MAX and AVG) obtain their best performances at the second level of the hierarchy. This emphasizes that the extraction of a hierarchy of clusters could provide some improvements with respect to a flat clustering. This is not so evident for HOCCLUS2 even if, analogously to LP-HCLUS, it is able to extract a hierarchy. The results in terms of AUTPR@*k*, AUROC and AUPR (see Table 3) confirm the superiority of LP-HCLUS_MAX over the competitors.

**Table 3:** AUTPR@k, AUROC and AUPR values for the dataset HMDD, obtained with the best configuration (*α*=0.2,*β*=0.4) at different levels of the hierarchy

		AUTPR@k	AUPR	AUROC
LP-HCLUS-NoLP		0.000000	0.000000	0.496169
ncPred		0.087370	0.007540	0.584268
LP-HCLUS AVG	*Level 1*	0.042658	0.005437	0.523872
	*Level 2*	0.056392	0.003140	0.548665
	*Level 3*	0.020129	0.000469	0.515470
LP-HCLUS MAX	*Level 1*	0.088130	0.010865	0.568056
	*Level 2*	**0.109292**	**0.013420**	**0.585560**
	*Level 3*	0.104244	0.011983	0.580824
LP-HCLUS MIN	*Level 1*	0.031888	0.001935	0.519936
	*Level 2*	0.032765	0.001232	0.524077
	*Level 3*	0.011012	0.000170	0.505846
LP-HCLUS EC	*Level 1*	0.005626	0.000035	0.501872
	*Level 2*	0.004851	0.000030	0.500943
	*Level 3*	0.006493	0.000050	0.502762
HOCCLUS2 AVG	*Level 1*	0.018339	0.000839	0.511722
	*Level 2*	0.016484	0.000670	0.509663
	*Level 3*	0.012082	0.000287	0.507020
HOCCLUS2 MAX	*Level 1*	0.018332	0.000829	0.510398
	*Level 2*	0.016065	0.000659	0.508897
	*Level 3*	0.011150	0.000274	0.506331
HOCCLUS2 MIN	*Level 1*	0.015922	0.000753	0.509336
	*Level 2*	0.016401	0.000668	0.509542
	*Level 3*	0.011647	0.000270	0.506575
HOCCLUS2 EC	*Level 1*	0.013922	0.000536	0.507314
	*Level 2*	0.013717	0.000352	0.507112
	*Level 3*	0.013065	0.000253	0.507751

The results in terms of AUPR and AUROC values can only be used for relative comparison and not as absolute evaluation measures because they are spoiled by the assumption made on unknown associations, that are considered as negative examples

The best result is highlighted in boldface.

### Results - ID dataset

**Fig. 14:**
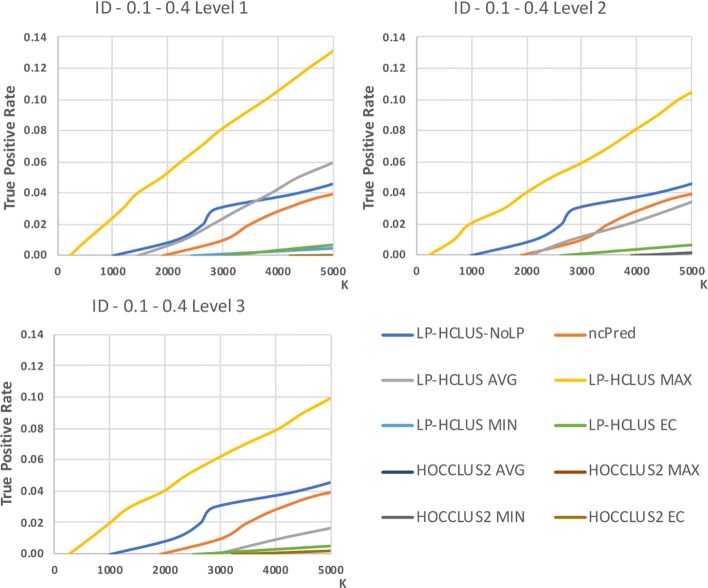
TPR@*k* results for the dataset ID, obtained with the best configuration (*α*=0.1,*β*=0.4) at different levels of the hierarchy

**Fig. 15:**
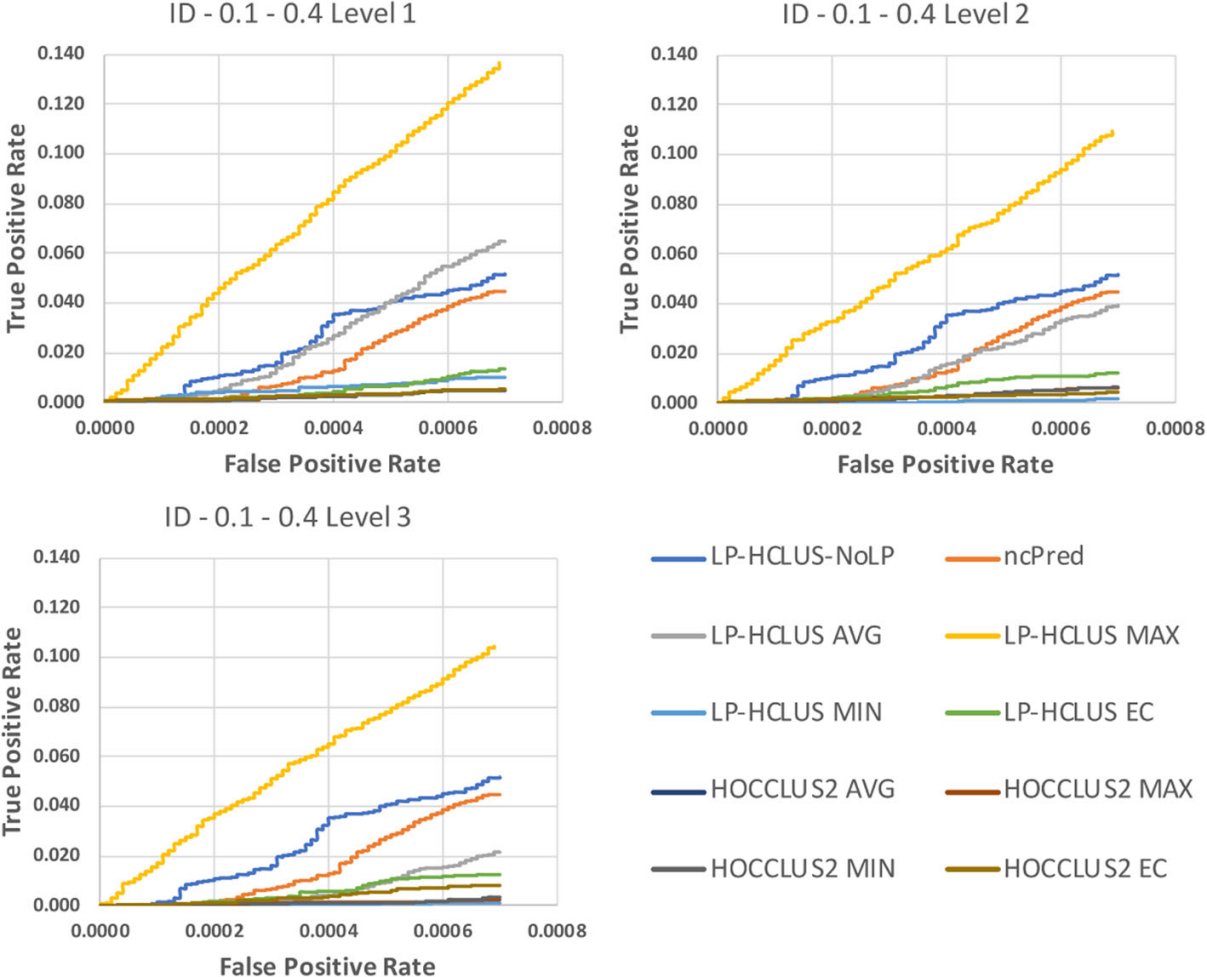
ROC curves for the dataset ID, obtained with the best configuration (*α*=0.1,*β*=0.4) at different levels of the hierarchy. These curves can only be used for relative comparison and not as absolute evaluation measures because they are spoiled by the assumption made on unknown relationships

**Fig. 16:**
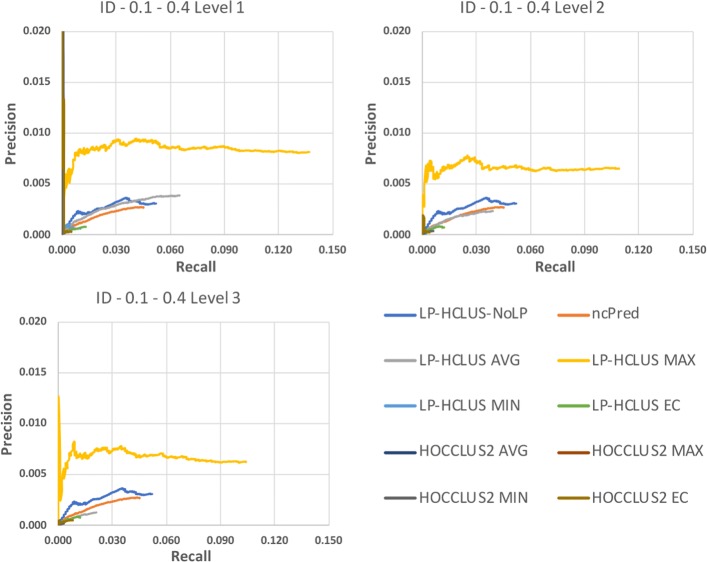
Precision-Recall curves for the dataset ID, obtained with the best configuration (*α*=0.1,*β*=0.4) at different levels of the hierarchy. These curves can only be used for relative comparison and not as absolute evaluation measures because they are spoiled by the assumption made on unknown relationships

In Figures 14, 15 and 16 we show the results obtained on the Integrated Dataset (ID) in terms of TPR@k, ROC and Precision-Recall curves, while in Table 4, we report the AUTPR@k, AUROC and AUPR values. It is noteworthy that this dataset is much more complex than HMDD, because it consists of several types of nodes, each associated with its attributes. In this case, the system LP-HCLUS can fully exploit information brought by other node types to predict new associations between ncRNAs and diseases.

**Table 4:** AUTPR@k, AUROC and AUPR values for the dataset ID, obtained with the best configuration (*α*=0.1,*β*=0.4) at different levels of the hierarchy

		AUTPR@k	AUPR	AUROC
LP-HCLUS-NoLP		0.024087	0.000150	0.525501
ncPred		0.015365	0.000087	0.521975
LP-HCLUS AVG	*Level 1*	0.024335	0.000198	0.532059
	*Level 2*	0.013660	0.000080	0.519290
	*Level 3*	0.005883	0.000024	0.510396
LP-HCLUS MAX	*Level 1*	**0.070639**	**0.001218**	**0.567991**
	*Level 2*	0.054821	0.000780	0.554388
	*Level 3*	0.055141	0.000756	0.551873
LP-HCLUS MIN	*Level 1*	0.005451	0.000010	0.504690
	*Level 2*	0.000474	0.000000	0.500490
	*Level 3*	0.000305	0.000000	0.500154
LP-HCLUS EC	*Level 1*	0.004609	0.000010	0.506366
	*Level 2*	0.005605	0.000013	0.505695
	*Level 3*	0.005353	0.000010	0.505862
HOCCLUS2 AVG	*Level 1*	0.002246	0.000087	0.502169
	*Level 2*	0.002553	0.000006	0.502843
	*Level 3*	0.000885	0.000001	0.501328
HOCCLUS2 MAX	*Level 1*	0.002238	0.000087	0.502169
	*Level 2*	0.002659	0.000005	0.502676
	*Level 3*	0.000973	0.000001	0.500826
HOCCLUS2 MIN	*Level 1*	0.002247	0.000087	0.502169
	*Level 2*	0.002553	0.000006	0.502843
	*Level 3*	0.000885	0.000001	0.501328
HOCCLUS2 EC	*Level 1*	0.002763	0.000015	0.502337
	*Level 2*	0.002320	0.000008	0.501835
	*Level 3*	0.003533	0.000007	0.503683

The results in terms of AUPR and AUROC values can only be used for relative comparison and not as absolute evaluation measures because they are spoiled by the assumption made on unknown associations, that are considered as negative examples

The best result is highlighted in boldface.

As it can be observed from the figures, thanks to such an ability, LP-HCLUS clearly outperforms all the competitors. It is noteworthy that also the simpler version of LP-HCLUS, i.e., LP-HCLUS-NoLP, is able to outperform the competitors, since it exploits the exploration of the network based on meta-paths. However, when we exploit the full version of LP-HCLUS, which bases its prediction on the clustering results, the improvement over the existing approaches becomes much more evident. These conclusions are also confirmed by the AUTPR@k, AUROC and AUPR values shown in Table 4.

### Statistical comparisons

By observing the results reported in Figs. 11, 12, 13, 14, 15 and 16, it is clear that the adoption of the Maximum (MAX) as LP-HCLUS aggregation function leads to the best results. This behavior can be motivated by the fact that such an approach rewards the associations which show at least one strong evidence from the clusters. Although such a behavior should be observed also with the Evidence Combination (EC) function, it is noteworthy that the latter also rewards associations which are confirmed by several clusters, even if they show a weak confidence. In this way, EC is prone to false positives introduced by the combined contribution of several weak relationships.

**Fig. 17:**
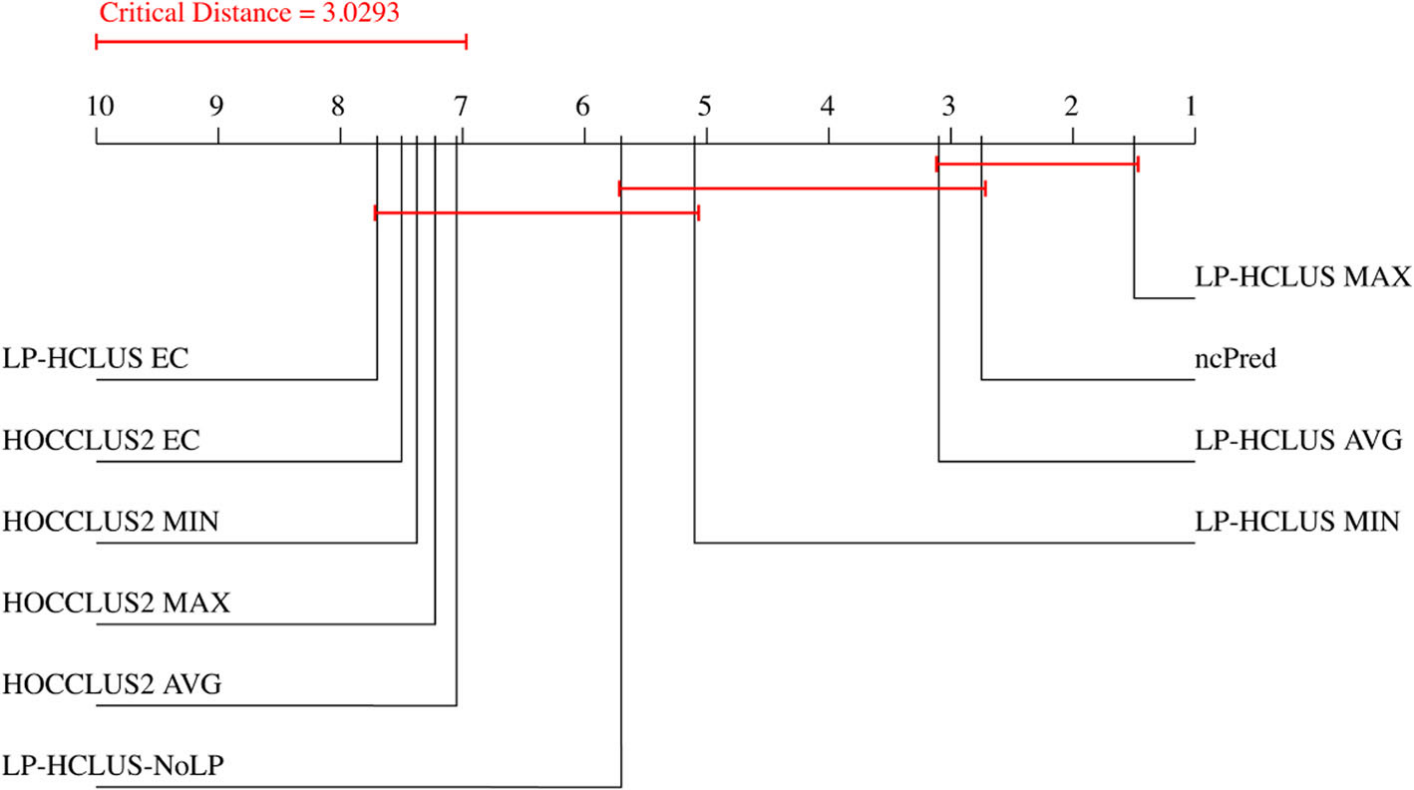
Result of the Friedman test with Nemenyi post-hoc test, with a significance level of 0.05, performed on the area under the TPR@*k* curve

In order to confirm the superiority of LP-HCLUS_MAX from a statistical viewpoint, we performed a Friedman test with Nemenyi post-hoc test with significance value of 0.05. This test is applied to the Area Under the TPR@*k* curve, in order to provide a *k*-independent evaluation of the results. By observing the results in Fig. 17, it is clear that LP-HCLUS_MAX is the best ranked method among the considered approaches. Since, at a glance, the difference between LP-HCLUS_MAX and ncPred is clear, but does not appear to be statistically significant with a test that evaluates differences across multiple systems, we performed three pairwise Wilcoxon tests (one for each hierarchical level), with the Bonferroni correction. In this way, it is possible to directly compare LP-HCLUS_MAX and ncPred. Looking at the average Area Under the TPR@*k* and *p*-values reported in Table 5, it is clear that the difference between LP-HCLUS_MAX and its direct competitor ncPred is large (especially for the ID dataset) and, more importantly, statistically significant for all the hierarchical levels, at a significance value of 0.01.

**Table 5:** Average Area Under the TPR@*k* curve and *p*-values obtained by the Wilcoxon signed-rank test with the Bonferroni correction

	Average Area Under TPR@*k*		*p*-values
Method	HMDD v3.0 dataset	ID dataset	LP-HCLUS vs ncPred
ncPred	0.087370	0.015365	
LP-HCLUS_MAX_L1	0.088130	**0.070639**	0.005833 (+)
LP-HCLUS_MAX_L2	**0.109292**	0.054821	0.000266 (+)
LP-HCLUS_MAX_L3	0.104244	0.055141	0.000266 (+)

The best result for each dataset is emphasized in boldface. (+) indicates that LP-HCLUS significantly outperforms ncPred (*p*-value <0.01)

The best result is highlighted in boldface.

## Discussion

In this section we discuss about the results of the comparison of LP-HCLUS with its competitors from a qualitative viewpoint, in order to assess the validity of the proposed system as a useful tool for biologists.

### Discussion on the HMDD v3 dataset

**Fig. 18:**
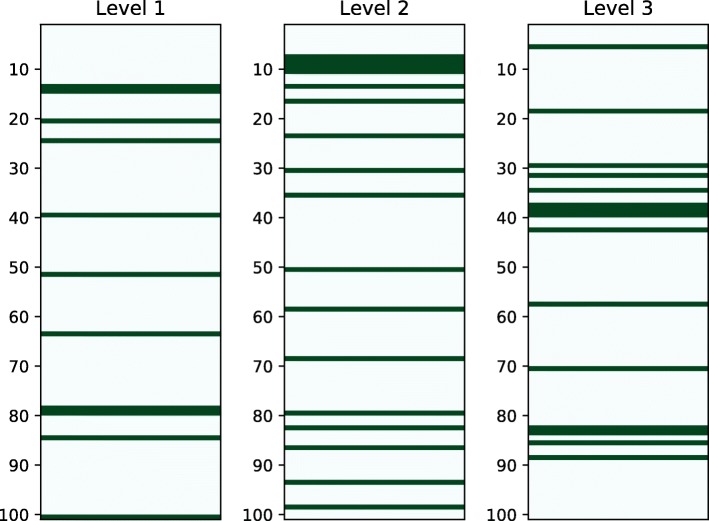
A graphical representation of the top-100 relationships predicted by LP-HCLUS from HMDD v3.0. The dark green lines represent the position of the relationships that have been subsequently validated and introduced in HMDD v3.2

We performed a comparative analysis between the results obtained by LP-HCLUS against the validated interactions reported in the updated version of HMDD (i.e., v3.2 released on March 27th, 2019). A graphical overview of the results of this analysis is provided in Fig. 18, while the detailed results are provided in Additional file 3, where the relationships introduced in the new release of HMDD are highlighted in green. The general conclusion we can draw from Fig. 18 is that several relationships predicted by LP-HCLUS have been introduced in the new HMDD release v3.2.

In particular, we found 3055 LP-HCLUS predictions confirmed by the new release of HMDD at the hierarchy level 1 (score range 0.97-0.44), 4119 at level 2 (score range 0.93-0.37) and 4797 at level 3 (score range 0.79-0.37). Overall, these results underline the behavior of LP-HCLUS at the different levels of the hierarchy. As expected, the number of predictions grows progressively from the lowest to the highest levels of the hierarchy, due to the less stringent constraints imposed by the algorithm, that allow LP-HCLUS to identify larger clusters at higher levels of the hierarchy. Larger clusters, even if possibly less reliable, in some cases can lead to the identification of less obvious functional associations.

Comparing the diseases at different levels of the hierarchy confirmed in the updated release of HMDD, we found associations involving 276 diseases at level 1, 360 at level 2 and 395 at level 3. Among the diseases involved in new associations predicted at level 3, but not at levels 1 and 2, there is the *acquired immunodeficiency syndrome*, a chronic, potentially life-threatening condition caused by the human immunodeficiency virus (HIV). The associations predicted by LP-HCLUS for this disease, confirmed in HMDD v3.2, involve hsa-mir-150 (with score 0.68) and hsa-mir-223 (with score 0.63). Such associations have been reported in [36]. The authors show the results of a study where the regulation of cyclin T1 and HIV-1 replication has been evaluated in resting and activated CD4+ T lymphocytes with respect to the expression of endogenous miRNAs. In this study, the authors demonstrated that miR-27b, miR-29b, miR-150, and miR-223 are significantly downregulated upon CD4(+) T cell activation, and identified miR-27b as a novel regulator of cyclin T1 protein levels and HIV-1 replication, while miR-29b, miR-223, and miR-150 may regulate cyclin T1 indirectly.

**Fig. 19:**
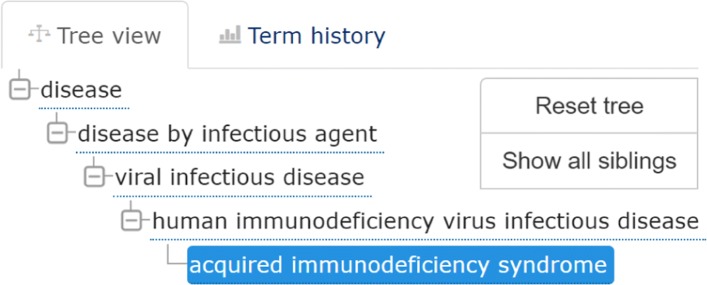
Ontology classification of *acquired immunodeficiency syndrome* according to EMBL-EBI Ontology Lookup Service [37]

Other validated miRNAs associated with the *acquired immunodeficiency syndrome* in HMDD v3.2 are hsa-mir-27b, -29b, -29a, -29b-1 and hsa-mir-198. As shown in Fig. 19, these miRNAs, although not directly associated by LP-HCLUS with the *acquired immunodeficiency syndrome*, have been associated with disease terms strictly related to the immune system, with a score and specificity depending on the hierarchy level. In particular, at level 1, they have been associated with the *immune system disease* term (DOID_2914, a subclass of *disease of anatomical entity*) with a score ranging from 0.48 for hsa-mir-29b to a maximum value of 0.67 for hsa-mir-29a. At level 2 of the hierarchy, in addition to the classification in the *immune system disease*, they have also been associated with the *human immunodeficiency virus infection* (DOID_526) that is a subclass of *viral infectious disease* (DOID_934) and the direct parent of the *acquired immunodeficiency syndrome* (DOID_635). At level 3, all the miRNAs have also been associated with the *viral infectious disease* term.

In addition to hsa-mir-155 and hsa-mir-223, LP-HCLUS returned many other associations involving *acquired immunodeficiency syndrome* with a high score. In particular, 59 different miRNAs have been associated at level 2 (score between 0.74 and 0.63), and 191 at level 3 (score between 0.68 and 0.63). Considering such high scores, we investigated in the literature for some of the associated miRNAs. In particular, we searched for hsa-mir-30a, that was among the miRNAs with the highest association score (0.74 at the 2nd level) and found a work where it has been significantly associated with other six miRNAs (i.e., miR-29a, miR-223, miR-27a, miR-19b, miR-151-3p, miR-28-5p, miR-766) as biomarker for monitoring the immune status of patients affected by *acquired immunodeficiency syndrome* [38].

Together with hsa-mir-30a, also other miRNAs belonging to the same family (i.e., hsa-mir-30b, -30c and -30e) have been associated by LP-HCLUS with the same disease. In [39], four miRNA-like sequences (i.e., hsa-mir-30d, hsa-mir-30e, hsa-mir-374a and hsa-mir-424) were identified within the env and the gag-pol encoding regions of several HIV-1 strains. The mapping of their sequences within the HIV-1 genomes localized them to the functionally significant variable regions, designated V1, V2, V4 and V5, of the env glycoprotein gp120. This result was important because the regions V1 to V5 of HIV-1 envelopes contain specific and well-characterized domains that are critical for immune responses, virus neutralization and disease progression. The authors concluded that the newly discovered miRNA-like sequences in the HIV-1 genomes might have evolved to self-regulated survival of the virus in the host by evading the innate immune responses and therefore influencing persistence, replication or pathogenicity of the virus.

Another example of reliable associations of ncRNAs with the *acquired immunodeficiency syndrome* identified by LP-HCLUS, and not present in HMDD 3.2, are those with hsa-mir-125b, hsa-mir-28 and hsa-mir-382. These associations are confirmed in [40], where the authors provided evidence that these miRNAs can contribute, alongside hsa-mir-155 and hsa-mir-223, to the HIV latency. It is noteworthy that these associations appear only at level 3 of the hierarchy but not at levels 2 or 1.

Altogether, these results highlight two interesting features of LP-HCLUS: the ability to discover meaningful functional associations, and the way the hierarchical clustering can help in the identification of hidden information. In principle, none of the hierarchy levels should be ignored. As shown for the case of the *acquired immunodeficiency syndrome*, the first hierarchical level, although in principle more reliable (since based on more stringent constraints), in some cases is not able to capture less obvious existing associations. On the other hand, results obtained from higher levels of the hierarchy are much more inclusive and can provide pieces of information that, in the lowest levels, are hidden, and that can be pivotal to the specific aims of a research investigation.

Finally, we compared the ranking values assigned by LP-HCLUS, ncPred and HOCCLUS2 on the same associations, that are, those confirmed in the HMDD v3.2 release (see Additional file 5). At this purpose, we computed the AUTPR@*k* by considering the new interactions introduced in HMDD v3.2 as ground truth. By observing the results reported in Table 6, we can confirm that LP-HCLUS based on the MAX measure outperforms all the competitors in identifying new interactions from the previous version of the dataset (HMDD v3.0) that have been subsequently validated and introduced in the latest version (HMDD v3.2).

**Table 6:** AUTPR@k computed using the new associations introduced in the new version of HMDD v3.2 as ground truth

		AUC TPR@k
LP-HCLUS-NoLP		0.00000
ncPred		0.01448
LP-HCLUS AVG	*Level 1*	0.01754
	*Level 2*	0.02663
	*Level 3*	0.01453
LP-HCLUS MAX	*Level 1*	0.03247
	*Level 2*	**0.03423**
	*Level 3*	0.03111
LP-HCLUS MIN	*Level 1*	0.01846
	*Level 2*	0.02197
	*Level 3*	0.00962
LP-HCLUS EC	*Level 1*	0.00695
	*Level 2*	0.00527
	*Level 3*	0.00548
HOCCLUS2 AVG	*Level 1*	0.01750
	*Level 2*	0.00627
	*Level 3*	0.00962
HOCCLUS2 MAX	*Level 1*	0.01774
	*Level 2*	0.00763
	*Level 3*	0.00991
HOCCLUS2 MIN	*Level 1*	0.01657
	*Level 2*	0.00627
	*Level 3*	0.00962
HOCCLUS2 EC	*Level 1*	0.01689
	*Level 2*	0.01269
	*Level 3*	0.01252

The best result is highlighted in boldface.

### Discussion on the integrated dataset

As concerns the ID dataset, we performed a qualitative analysis of the top-ranked relationships predicted by LP-HCLUS, i.e., on those with a score equal to 1.0. For this purpose, we exploited MNDR v2.0 [41], which is a comprehensive resource including more than 260,000 experimental and predicted ncRNA-disease associations for mammalian species, including lncRNA, miRNA, piRNA, snoRNA and more than 1,400 diseases. Data in MNDR comes from manual literature curation and other resources, and include a confidence score for each ncRNA–disease association. Experimental evidences are manually classified as *strong* or *weak*, while the confidence score is calculated according to the evidence type (*s*: strong experimental evidence, *w*: weak experimental evidence, *p*: prediction) and the number of evidences.

The top-ranked relationships returned by LP-HCLUS involve 1,067 different diseases and 814 different ncRNAs, consisting of 488 miRNAs and 326 lncRNAs, among which there are several antisense RNAs and miRNA hosting genes. Table 7 shows some examples of top-ranked interactions predicted by LP-HCLUS and involving 4 ncRNAs, i.e., h19, wrap53, pvt1 and hsa-miR-106b.

**Table 7:** Examples of top-ranked ncRNA-disease associations predicted by LP-HCLUS with a score equal to 1.0

ncRNA	Disease
h19	bone diseases, developmental
h19	carcinoma, hepatocellular
h19	colorectal neoplasms
h19	liver neoplasms
h19	parkinson disease, secondary
hsa-miR-106b	aging, premature
hsa-miR-106b	burkitt s lymphomas
hsa-miR-106b	disease progression
pvt1	aging, premature
pvt1	disease progression
wrap53	adrenal gland neoplasms
wrap53	adrenocortical carcinoma
wrap53	emphysema

h19 is a long intergenic ncRNA (lincRNA) and a developmentally-regulated maternally-imprinted gene that is expressed only from the inherited chromosome 11. A putative function assigned to it is a tumor suppressor activity. GeneCards (GCID:GC11M001995) reports its association with the Wilms Tumor 2 (WT2) and Beckwith-Wiedemann Syndrome, both caused by mutation or deletion of imprinted genes within the chromosome 11p15.5 region. Other sources, such as GenBank [42] and MNDR [41, 43], report the association of h19 with many other human diseases, the majority being different types of tumors.

Searching for h19-disease associations in MNDR, we obtained 101 results with a confidence score ranging from 0.9820 to 0.1097. The same search performed on the output produced by LP-HCLUS (0.1 - 0.4, first level of the hierarchy) returned 993 associations with a score ranging from 1.0 to 0.4. A comparative analysis of the results shows a perfect match of 33 predictions (see Table 8), many of which also with a similar confidence score, despite the different approaches adopted to calculate them.

**Table 8:** Result of matching between the associations predicted by LP-HCLUS and those present in MNDR

ncRNA	Disease	LP-HCLUS	MNDR
h19	adenocarcinoma	0.7455674	s: 0.7311
h19	adrenocortical carcinoma	0.8150848	s: 0.7311
h19	aortic valve disease	0.6492379	s: 0.7311
h19	astrocytoma	0.7455674	s: 0.7311
h19	breast adenocarcinoma	0.7005121	s: 0.7311
h19	carcinoma, non-small-cell lung	0.7052352	s: 0.9820, p: 0.1097
h19	chronic myeloid leukemia	0.7005121	s: 0.8808
h19	colon carcinoma	0.7005121	s: 0.8589
h19	colorectal cancer	0.8150848	s: 0.9820, p: 0.1097
h19	coronary artery disease	0.6600133	w: 0.4752
h19	embryonal carcinoma	0.6522726	s: 0.9526
h19	endometriosis	0.7052352	s: 0.8808
h19	esophageal cancer	0.8150848	s: 0.8589
h19	gallbladder cancer	0.6522726	s: 0.8808
h19	heart defects, congenital	0.6703589	s: 0.8589
h19	laryngeal squamous cell carcinoma	0.6522726	s: 0.9526
h19	liver neoplasms	1.0000000	w: 0.4752
h19	lung adenocarcinoma	0.6669160	s: 0.8589
h19	lymphoma	0.6962170	p: 0.1321
h19	osteoarthritis	0.6749659	w: 0.4752
h19	osteosarcoma	0.7732385	s: 0.9820
h19	ovarian neoplasms	0.7052352	s: 0.8589, p: 0.1097
h19	pancreatic cancer	0.8150848	s: 0.8808
h19	pancreatic ductal adenocarcinoma	0.6575157	s: 0.9526
h19	polycythemia vera	0.7005121	s: 0.7311
h19	prostatic neoplasms	0.7052352	s: 0.7311, p: 0.1097
h19	rheumatoid arthritis	0.6703589	s: 0.9526
h19	schizophrenia	0.7052352	p: 0.1097
h19	squamous cell carcinoma	0.6826756	w: 0.4752
h19	thyroid cancer	0.7732385	s: 0.8808, p: 0.1097
h19	urinary bladder neoplasms	0.6962170	p: 0.1097
h19	uterine cervical neoplasms	0.7455674	s: 0.7311, p: 0.1097

MNDR scores are associated with an evidence type: *s*: strong experimental evidence, *w*: weak experimental evidence, *p*: prediction

Among the top-ranked associations predicted by LP-HCLUS involving h19, the association with “bone diseases, developmental” is not present in the results obtained by the MNDR database (see Table 7). Bone diseases can have different origins and can be also related to hyperfunction or hypofunction of the endocrine glands, such as pituitary gland, thyroid gland, parathyroid glands, adrenal glands, pancreas, gonads, and pineal gland. The results of the comparative analysis with the data in MNDR, in addition to the relationship with osteosarcoma (LP-HCLUS score 0.7732385; MNDR confidence score s: 0.9820) show associations between h19 and other diseases which involve endocrine glands such as: ovarian neoplasms (LP-HCLUS score 0.7052352; MNDR confidence score p: 0.1097, s: 0.8589); pancreatic cancer (LP-HCLUS score 0.8150848; MNDR confidence score s: 0.8808); pancreatic ductal adenocarcinoma (LP-HCLUS score 0.6575157; MNDR confidence score s: 0.9526) and thyroid cancer (LP-HCLUS score 0.7732385; MNDR confidence score s: 0.8808, p: 0.1097) (See Table 8). This indicates that h19 can have a relationship with endocrine glands functions and, therefore, can be related to bone diseases as predicted by LP-HCLUS.

## Conclusions

In this paper, we have tackled the problem of predicting possibly unknown ncRNA-disease relationships. The approach we proposed, LP-HCLUS, is able to take advantage from the possible heterogeneous nature of the attributed biological network analyzed. In this way, it is possible to identify ncRNA-disease relationships by taking into account the properties of additional biological entities (e.g. microRNAs, lncRNAs, target genes) they are connected to.

Methodologically, LP-HCLUS is based on the identification of paths in the heterogeneous attributed biological network, which potentially confirm the connection between a ncRNA and a disease, and a clustering phase, which is preparatory to a link prediction phase. In this way, it is possible to catch the network autocorrelation phenomena and exploit information implicitly conveyed by the network structure.

The results confirm the initial intuitions and show competitive performances of LP-HCLUS in terms of accuracy of the predictions, also when compared, through a statistical test (at a significance level of 0.01), with state-of-the-art competitor systems. These results are also supported by a comparison of LP-HCLUS predictions with data reported in MNDR and by a qualitative analysis that revealed that several ncRNA-disease associations predicted by LP-HCLUS have been subsequently experimentally validated and introduced in a more recent release (v3.2) of HMDD.

Finally, the association between the long-intergenic ncRNA h19 and bone diseases, predicted by LP-HCLUS, suggests an important functional role of h19 in the regulation of endocrine glands functions. This further confirms the potential of LP-HCLUS as a prediction tool for the formulation of new biological hypothesis and experimental validations for the characterization of the roles of ncRNAs in biological processes.

For future work, we plan to extend our approach in order to predict the direction of the relationships, and not only their presence. This would require to identify and deal with cause/effect phenomena. Depending on the availability of data, it would also be very interesting to evaluate the results of LP-HCLUS analysis on tissue-specific datasets or on datasets related to physiological or pathological specific conditions.

## Supplementary information

**Additional file 1** Discussion of related work.

**Additional file 2** Analysis of the time complexity of lP-HCLUS.

**Additional file 3** Complete results of the comparative analysis between the predictions returned by lP-HCLUS from hMDD v3.0 and the new validated relationships in hMDD v3.2.

**Additional file 4** Detailed list of associations involving the *acquired immunodeficiency syndrome* and similar disease terms in three hierarchical levels extracted by lP-HCLUS.

**Additional file 5** Comparative analysis of the ranking produced by lP-HCLUS and its competitors with respect to the new validated relationships in hMDD v3.2.

## Data Availability

The system LP-HCLUS, the adopted datasets and all the results are available at: http://www.di.uniba.it/ extasciitildegianvitopio/systems/lphclus/

## Abbreviations

AUPR: Area under the Precision-Recall curve
AUROC: Area under the ROC curve
AUTPR@k: Area under the TPR@k curve
AVG: Average
CUI: Concept Unique Identifier
DOID: Human Disease Ontology ID
EC: Evidence Combination
EMBL-EBI: European Molecular Biology Laboratory - European Bioinformatics Institute
GBA: Guilt-By-Association principle
GCID: GeneCards ID
HOCCLUS2: Hierarchical Overlapping Co-CLUStering2
HPO: Human Phenotype Ontology
lncRNA: long non-coding RNA
LP-HCLUS: Link Prediction through Hierarchical CLUStering
MAX: Maximum
MeSH: Medical Subject Headings
MIN: Minimum
miRNA: microRNA
ncRNA: non-coding RNA
OMIM: Online Mendelian Inheritance in Man
RefSeq: NCBI’s Reference Sequences database
RNA: RiboNucleic Acid
ROC: Receiver Operating Characteristic
SNP: Single-Nucleotide Polymorphism
TPR@k: True Positive Rate at k
UML: Unified Modeling Language
UMLS: Unified Medical Language System

## Footnotes

1 http://www.cuilab.cn/lncrnadisease

2 Dataset “Data S3” in https://www.sciencedirect.com/science/article/pii/S009286741300439X?via%3Dihub#mmc3

3 http://www.disgenet.org/

4 http://www.mir2disease.org/
