# Supplementary material for: Prediction of new associations between ncRNAs and diseases exploiting multi-type hierarchical clustering
Source: BMC Bioinformatics. 2020 Feb 24;21:70. doi: 10.1186/s12859-020-3392-2 (PMC7041288; doi:10.1186/s12859-020-3392-2)
Supplement: Supplementary file 1 — Additional file 1 Discussion of related work. [file 12859_2020_3392_MOESM1_ESM.pdf]

Additional File 1
Related Work

In this supplement, we briefly describe the related work in the different fields in which the paper finds its roots. In particular, we report a brief discussion of *i)* the existing methods for link prediction applied in other application domains, *ii)* approaches for biological network modeling and reconstruction, and *iii)* methods for the identification of new associations in the biological field, also between heterogeneous entities.

Link prediction methods

Several application domains can rely on network structures for the representation of their data. The identification/prediction of the existence of (previously unknown) links among objects is one of the most common tasks on network data, since it allows inferring new relationships among the considered entities, which discovery through real observation would require extensive resources.

Relevant example where link prediction approaches are extensively adopted are social networks [1, 2, 3, 4, 5], where the goal is to identify possible new friends or pages of interest, and recommender systems [6, 7, 8], which aim to suggest new items according to users' interests. Recently, this task has been extensively studied also in the biological field, where the goal is to infer possible functions of biological entities that are then validated experimentally (see the next subsection). More formally, given a network, the goal of the link prediction task is to compute the likelihood that an unknown link exists (see Figure 1). According to [9], we can identify four categories of link prediction approaches:

• *Similarity-based methods.* This category includes methods that exploit the computation of a similarity measure among nodes to predict new links. In particular, these methods are based on the assumption that similar nodes have higher probability to be linked. Formally, for each pair of nodes n_i, n_j, these methods compute the similarity $sim(n_i, n_j)$, and predict a new link between n_i

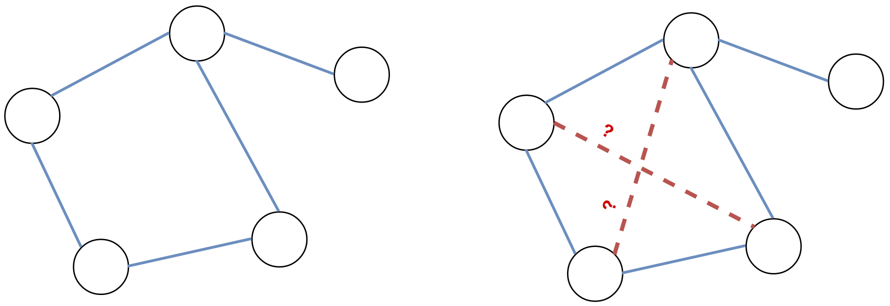

Figure 1: **Link Prediction task in a homogeneous network.** Given a network (on the left), the goal of the link prediction task is to identify possibly unknown relationships between nodes in the network.

and n_j if $sim(n_i, n_j)$ is greater than a pre-defined threshold. Multiple measures can be adopted, either based on topological information (e.g., the neighborhood of nodes) [3, 10, 11], on the features associated with nodes and links [12], or on a combination of them [4, 5].

• *Probabilistic and statistical methods.* Methods falling in this category aim at identifying a probabilistic model which best fits the network, and to exploit it to compute the likelihood of the existence of other unknown relationships [13, 14, 15].

• *Algorithmic methods.* These approaches include methods based on machine learning methods. In particular, they learn a classifier from a set of known examples of links, that is subsequently able to associate a class (true, false) or a probability (in $[0, 1]$) to unseen links. Different learning models have been adopted in the literature [16], including support vector machines (SVM) [17, 18], decision trees [19] and neural networks [20].

According to this classification, LP-HCLUS falls in the category of algorithmic methods, since it strongly relies on a clustering approach to predict new relationships and to associate them with a score in $[0, 1]$. Moreover, LP-HCLUS also adopts a similarity-based approach, since the first phase of the method is based on the computation of the similarities between target nodes, taking into account the paths in the network and the attributes of the nodes. For this reason, in the following we briefly discuss some existing algorithmic and similarity-based approaches.

In [1] the authors propose a prediction method that combines social network analysis and text mining to identify new links. In particular, the authors analyze a co-authorship network, where nodes represent authors and edges represent collaborations among them, aiming to predict whether two authors will co-author a paper in the future. Methodologically, the authors evaluate the adoption of a classifier based on neural networks and decisions trees.

A link prediction method for social networks is proposed in [2], where the goal is to identify new associations between users. As in [1], the link prediction task is treated as a binary classification task, where the classifier is based either on a single model (decision trees, neural networks or SVMs) or on ensembles.

In [6] the authors propose a link prediction method to improve the performance of a recommender system, by predicting new links between customers and products. The method falls in the category of similarity-based approaches and it is able to use both domain-based semantic similarity and topology features of the network to improve the quality of recommendations. Analogously, in [7] the authors focus on the design of a recommender system that exploits a network, where nodes represent users and edges represent transactions among them. This work exploits a similarity-based method, taking into account both the structure of the network and the attributes of the nodes.

In [8] the authors focus on the estimation of the user preferences about web pages, in order to predict the likelihood that a user will like a page in the future. The authors propose a hybrid method that initially acts as a similarity-

based approach, considering user-user similarity (based on the pages clicked by each user), page-page content similarity and page-page co-occurrence similarity. Then, in a second stage, the method learns a support vector machine able to distinguish pages that will be visited by a user from those that will not, according to their features.

Although some of these works are able to consider both the features of nodes and the structure of the network, they are commonly limited to homogeneous networks or to specific heterogeneous networks, consisting of a limited (i.e., pre-defined) number of node types or organized in a pre-defined structure. All these limitations are overcome by the proposed method LP-HCLUS, which provides a solution towards the identification of new links in heterogeneous attributed networks of arbitrary structure, consisting of multiple, interacting entities.

Modeling and reconstruction of biological networks

The working mechanisms in an organism are usually modelled as biological networks. The regulations modeled by such networks can include the control of transcription of DNA into mRNA (messenger RNA) or the translation of mRNAs into proteins [21, 22]. Such biological networks are typically referred to as Gene-Regulatory Networks (GRNs), where nodes represent molecular entities, such as transcription factors (TFs), proteins and metabolites, whereas edges represent interactions, such as protein-protein and protein-RNA interactions.

The reconstruction of the structure of such networks can be performed experimentally by using ChIP-chip or ChIP-sequencing [23], bacterial one-hybrid system [24] or protein-binding microarrays [25]. However, they are technically and financially demanding, and data-driven prediction approaches are usually preferred. A review of the literature shows the existence of a wide range of approaches for network reconstruction. Indeed, we can find a plethora of methods based on different approaches, such as relevance networks, Bayesian Networks, clustering, differential equations, probabilistic models, random walk processes, Markov chains and maximum likelihood (see [26] for an overview).

However, it has been proved that single prediction approaches do not consistently produce accurate results across all the datasets [27]. Therefore, several approaches aiming to post-process or combine the output of multiple prediction algorithms have recently been proposed in the literature. In particular, in the first category, we can find the methods ARACNE [28] and LOCANDA [29, 30], which main goal is to reduce false positive interactions introduced by reconstruction algorithms, due to the erroneous introduction of indirect relationships. Moreover, in [31] the authors propose a method that, starting from a protein-protein interaction network, is able to remove noisy edges and to suggest new promising interactions.

In the second category, we can find ensemble-based and meta-learning approaches. In [27], the authors propose to compute the average rank of each prediction, returned by multiple algorithms, namely the participants to the DREAM5 challenge. They proved that no single approach was able to outper-

form the ensemble on three different datasets. A more sophisticated solution for combining the output of several methods has been proposed in [32]. In this work, the predictions returned by each inference method are ranked according to their scores and the combined rank of each interaction is computed by taking the k-th highest rank among all the considered methods, where k is an input parameter.

In [33] the authors propose an ensemble-based approach which is able to combine the output of different link prediction algorithms. It works in a semi-supervised learning setting, i.e., it is able to exploit information conveyed from both labeled and unlabeled examples. Such a characteristic is very useful in the biological domain where the amount of positive, or labeled, examples is much lower than the number of unlabeled ones.

Finally, it is worth to mention that some recent works also exploited transfer learning approaches in order to improve the accuracy of the reconstruction. Contrary to the other methods, the improvement here is not achieved by considering a pool of methods, but rather multiple, related sources of data. A relevant example is the work [34], where the authors exploited data related to the mouse for the reconstruction of the human regulatory network in a positive-unlabeled semi-supervised setting.

Prediction of biological associations

In the literature, we can find several works aiming at predicting new associations between entities in the biological field. One example is the work in [35], where the goal is to identify associations among different diseases. This goal is achieved by adopting three different types of measures, i.e., annotation-based measure, function-based measure and topology-based measure, used to compute the similarity among diseases. Experimental results show that the proposed measures are able to discover new associations between diseases and that the predicted disease-disease associations are highly correlated with associations already known in the literature.

In [36] the authors adopt link prediction methods to solve the drug response problem, i.e., for the identification of relationships between drugs and cell lines. The idea is to consider a heterogeneous network composed by genes, drugs and cell lines and, for each drug-cell line pair, compute a score representing the likelihood that a given cell line is sensitive to a given drug. To reach this goal, for each pair, the method computes network profiles (a representation of the proximity of mutated genes in a cell line with respect to every other node in the network) using random walks with restart. Similarly, [37] proposes a new link prediction algorithm based on support vector regression and ridge regression, specifically for the prediction of cancer drug sensitivity, that is, to estimate the drug response to cancer. Both these algorithms are supervised, but they have different goals: the former is used to select better quality cancer cell lines, while the latter is exploited to select cancer cell lines and the top-k genes (i.e., features) using state-of-the-art CUR matrix decomposition.

The method presented in [38] predicts unknown drug-target interactions

(DTIs) by exploiting topological similarity information extracted by known DTIs. According to the empirical evaluation, this method, compared to previous approaches proposed for drug-target interaction prediction, appears to be effective also when there is a limited amount of information about the characteristics of drugs, targets or their interactions.

Recently, more attention has been paid on the study of ncRNAs. In [39] the authors analyze the associations between lncRNAs and diseases in order to understand the influence of this type of ncRNAs over complex diseases. The proposed method builds a bipartite graph based on known associations between diseases and genes and applies a propagation algorithm to discover hidden relationships between lncRNAs and diseases.

Another approach, called LION [40], constructs a tripartite network composed by lncRNAs, proteins and diseases. A random walk network diffusion algorithm is then applied to predict new lncRNA-disease associations exploiting the proximity of the lncRNA with respect to the disease, through their connections with proteins. Differently from other methods, LION is able to predict new associations without *a priori* lncRNA-disease information.

In [41] the authors proposed a new method, called *ncPred*, which is able to infer new associations between ncRNAs and diseases through the exploitation of recommendation techniques. In particular, ncPred exploits a tripartite graph, where nodes are ncRNAs, targets and diseases. The algorithm computes a weight for each ncRNA-disease pair by exploiting a multi-level resource transfer technique: new ncRNA-disease relationships are predicted by associating them through ncRNAs' targets. This approach is mainly limited by the low number of known ncRNA-target interactions and by the fact that it does not consider any biological information concerning each node (i.e., features possibly associated with ncRNAs and diseases) or their association (e.g., the type of the ncRNA-target interaction).

In [42] the authors proposed the algorithm HOCCLUS2, which extracts biclusters of microRNAs and gene transcripts, emphasizing functional relationships among them and, thus, possible new interactions. It can be easily extended to work with any kind of pairs of biological entities, such as ncRNAs and diseases, but does not exploit further possible features associated to them.

Most of the methods introduced above can potentially solve the problem of identifying new relationships between ncRNAs and diseases, although they require some adaptations. However, none of them simultaneously exhibits the ability of *i)* working on, and taking advantage from, arbitrarily-structured networks, consisting of an arbitrary number of types of nodes and links, and *ii)* exploiting the information conveyed by attributes, which describe specific properties of nodes in the attributed network.

References

[1] Bartal, A., Sasson, E., Ravid, G.: Predicting Links in Social Networks Using Text Mining and SNA. In: 2009 International Conference on

Advances in Social Network Analysis and Mining, pp. 131–136 (2009). doi:10.1109/ASONAM.2009.12

[2] Ahmed, C., ElKorany, A., Bahgat, R.: A supervised learning approach to link prediction in Twitter. Social Network Analysis and Mining **6**(1), 24 (2016). doi:10.1007/s13278-016-0333-1

[3] Srilatha, P., Manjula, R.: Structural similarity based link prediction in social networks using firefly algorithm. In: 2017 International Conference On Smart Technologies For Smart Nation (SmartTechCon), pp. 560–564 (2017). doi:10.1109/SmartTechCon.2017.8358434

[4] Yu, C., Zhao, X., An, L., Lin, X.: Similarity-based link prediction in social networks. J. Inf. Sci. **43**(5), 683–695 (2017). doi:10.1177/0165551516664039

[5] Prediction of missing links in social networks: Feature integration with node neighbour. Int. J. Web Based Communities **14**(1), 38–53 (2018). doi:10.1504/IJWBC.2018.090917

[6] Li, J., Zhang, L., Meng, F., Li, F.: Recommendation Algorithm based on Link Prediction and Domain Knowledge in Retail Transactions. Procedia Computer Science **31**, 875–881 (2014). doi:10.1016/j.procs.2014.05.339

[7] Bahabadi, M.D., Golpayegani, A.H., Esmaeili, L.: A Novel C2C E-Commerce Recommender System Based on Link Prediction: Applying Social Network Analysis. arXiv:1407.8365 [cs] (2014). arXiv: 1407.8365

[8] Sharif, M.A., Raghavan, V.V.: Link prediction based hybrid recommendation system using user-page preference graphs. In: 2017 International Joint Conference on Neural Networks (IJCNN), pp. 1147–1154 (2017). doi:10.1109/IJCNN.2017.7965981

[9] Martnez, V., Berzal, F., Cubero, J.-C.: A Survey of Link Prediction in Complex Networks. ACM Comput. Surv. **49**(4), 69–16933 (2016). doi:10.1145/3012704

[10] Jeh, G., Widom, J.: Simrank: A measure of structural-context similarity. In: Proceedings of the Eighth ACM SIGKDD International Conference on Knowledge Discovery and Data Mining. KDD '02, pp. 538–543. ACM, New York, NY, USA (2002). doi:10.1145/775047.775126. http://doi.acm.org/10.1145/775047.775126

[11] Zhou, T., Lü, L., Zhang, Y.-C.: Predicting missing links via local information. European Physical Journal B **71**, 623–630 (2009). doi:10.1140/epjb/e2009-00335-8. 0901.0553

[12] Jiang, M., Chen, Y., Chen, L.: Link Prediction in Networks with Nodes Attributes by Similarity Propagation. ArXiv e-prints (2015). 1502.04380

[13] Getoor, L., Friedman, N., Koller, D., Taskar, B.: Learning Probabilistic Models of Relational Structure. In: Proceedings of the Eighteenth International Conference on Machine Learning. ICML '01, pp. 170–177. Morgan Kaufmann Publishers Inc., San Francisco, CA, USA (2001). http://dl.acm.org/citation.cfm?id=645530.655682

[14] Getoor, L., Friedman, N., Koller, D., Taskar, B.: Learning Probabilistic Models of Link Structure. J. Mach. Learn. Res. **3**, 679–707 (2003)

[15] Popescul, A., Popescul, R., Ungar, L.H.: Statistical Relational Learning for Link Prediction, (2003)

[16] Hasan, M.A., Chaoji, V., Salem, S., Zaki, M.: Link prediction using supervised learning. In: In Proc. of SDM 06 Workshop on Link Analysis, Counterterrorism and Security (2006)

[17] Moradabadi, B., Meybodi, M.R.: Link prediction in fuzzy social networks using distributed learning automata. Applied Intelligence **47**(3), 837–849 (2017). doi:10.1007/s10489-017-0933-0

[18] Nguyen-Thi, A.-T., Nguyen, P.Q., Ngo, T.D., Nguyen-Hoang, T.-A.: Transfer adaboost svm for link prediction in newly signed social networks using explicit and pnr features. Procedia Computer Science **60**, 332–341 (2015). doi:10.1016/j.procs.2015.08.135

[19] de S, H.R., Prudncio, R.B.C.: Supervised link prediction in weighted networks. In: The 2011 International Joint Conference on Neural Networks, pp. 2281–2288 (2011). doi:10.1109/IJCNN.2011.6033513

[20] Shu, J., Chen, Q., Liu, L., Xu, L.: A link prediction approach based on deep learning for opportunistic sensor network. International Journal of Distributed Sensor Networks **13**(4) (2017). doi:10.1177/1550147717700642

[21] Penfold, C.A., Wild, D.L.: How to infer gene networks from expression profiles, revisited. Interface Focus **1**(6), 857–870 (2011)

[22] Atias, N., Sharan, R.: Comparative analysis of protein networks: hard problems, practical solutions. Communications of the ACM **55**(5), 88–97 (2012)

[23] Park, P.J.: ChIP–seq: Advantages and challenges of a maturing technology. Nature Reviews Genetics **10**(10), 669–680 (2009)

[24] Bulyk, M.L.: Discovering DNA regulatory elements with bacteria. Nature Biotechnology **23**(8), 942–944 (2005)

[25] Berger, M.F., Bulyk, M.L.: Universal protein-binding microarrays for the comprehensive characterization of the DNA-binding specificities of transcription factors. Nature Protocols **4**(3), 393–411 (2009)

[26] Lu, L., Zhou, T.: Link Prediction in Complex Networks: A Survey. Physica A: Statistical Mechanics and its Applications **390** (2011)

[27] Marbach, D., Costello, J.C., Kffner, R., Vega, N., Prill, R.J., Camacho, D.M., Allison, K.R., Kellis, M., Collins, J.J., Stolovitzky, G.: Wisdom of crowds for robust gene network inference. Nature methods **9**(8), 796–804 (2012). doi:10.1038/nmeth.2016

[28] Margolin, A.A., Nemenman, I., Basso, K., Wiggins, C., Stolovitzky, G., Favera, R.D., Califano, A.: ARACNE: An Algorithm for the Reconstruction of Gene Regulatory Networks in a Mammalian Cellular Context. BMC Bioinformatics **7**(Suppl 1), 7 (2006). doi:10.1186/1471-2105-7-S1-S7

[29] Pio, G., Ceci, M., Prisciandaro, F., Malerba, D.: LOCANDA: Exploiting Causality in the Reconstruction of Gene Regulatory Networks. In: Yamamoto, A., Kida, T., Uno, T., Kuboyama, T. (eds.) Discovery Science, pp. 283–297. Springer, Cham (2017)

[30] Pio, G., Ceci, M., Prisciandaro, F., Malerba, D.: Exploiting causality in gene network reconstruction based on graph embedding. Machine Learning (2019). doi:10.1007/s10994-019-05861-8

[31] Lei, C., Ruan, J.: A novel link prediction algorithm for reconstructing protein-protein interaction networks by topological similarity. Bioinformatics (Oxford, England) **29**(3), 355–364 (2013). doi:10.1093/bioinformatics/bts688

[32] Hase, T., Ghosh, S., Yamanaka, R., Kitano, H.: Harnessing diversity towards the reconstructing of large scale gene regulatory networks. PLoS Computational Biology **9**(11), 1003361 (2013)

[33] Pio, G., Malerba, D., D'Elia, D., Ceci, M.: Integrating microRNA target predictions for the discovery of gene regulatory networks: a semi-supervised ensemble learning approach. BMC bioinformatics **15 Suppl 1**, 4 (2014). doi:10.1186/1471-2105-15-S1-S4

[34] Mignone, P., Pio, G., DElia, D., Ceci, M.: Exploiting transfer learning for the reconstruction of the human gene regulatory network. Bioinformatics (2019). doi:10.1093/bioinformatics/btz781

[35] Sun, K., Gonalves, J.P., Larminie, C., Prulj, N.: Predicting disease associations via biological network analysis. BMC Bioinformatics **15**(1), 304 (2014). doi:10.1186/1471-2105-15-304

[36] Stanfield, Z., Cokun, M., Koyutrk, M.: Drug Response Prediction as a Link Prediction Problem. Scientific Reports **7**, 40321 (2017). doi:10.1038/srep40321

[37] Turki, T., Wei, Z.: A link prediction approach to cancer drug sensitivity prediction. BMC Systems Biology **11**(5), 94 (2017). doi:10.1186/s12918-017-0463-8

[38] Lu, Y., Guo, Y., Korhonen, A.: Link prediction in drug-target interactions network using similarity indices. BMC Bioinformatics **18** (2017). doi:10.1186/s12859-017-1460-z

[39] Yang, X., Gao, L., Guo, X., Shi, X., Wu, H., Song, F., Wang, B.: A Network Based Method for Analysis of lncRNA-Disease Associations and Prediction of lncRNAs Implicated in Diseases. PLOS ONE **9**(1), 87797 (2014). doi:10.1371/journal.pone.0087797

[40] Sumathipala, M., Maiorino, E., Weiss, S.T., Sharma, A.: Network Diffusion Approach to Predict LncRNA Disease Associations Using Multi-Type Biological Networks: LION. Frontiers in Physiology **10** (2019). doi:10.3389/fphys.2019.00888

[41] Alaimo, S., Giugno, R., Pulvirenti, A.: ncPred: ncRNA-Disease Association Prediction through Tripartite Network-Based Inference. Frontiers in Bioengineering and Biotechnology **2** (2014). doi:10.3389/fbioe.2014.00071

[42] Pio, G., Ceci, M., D'Elia, D., Loglisci, C., Malerba, D.: A Novel Biclustering Algorithm for the Discovery of Meaningful Biological Correlations between microRNAs and their Target Genes. BMC Bioinformatics **14**(Suppl 7), 8 (2013). doi:10.1186/1471-2105-14-S7-S8
