# Supplementary material for: Prediction of new associations between ncRNAs and diseases exploiting multi-type hierarchical clustering
Source: BMC Bioinformatics. 2020 Feb 24;21:70. doi: 10.1186/s12859-020-3392-2 (PMC7041288; doi:10.1186/s12859-020-3392-2)
Supplement: Supplementary file 2 — Additional file 2 Analysis of the time complexity of lP-HCLUS. [file 12859_2020_3392_MOESM2_ESM.pdf]

Additional File 2

Time Complexity Analysis

In this supplement, we show the result of the analysis of the LP-HCLUS time complexity by taking into account the complexity of each single phase, that are, the estimation of the strength of relationships between ncRNAs and diseases, the construction of the hierarchy of clusters and the prediction of new ncRNA-disease relationships.

For the estimation of the strength of relationships between ncRNAs and diseases, we assume that:

- nodes are equally distributed among types, i.e., $|V_1|$=$|V_2|$= ... =$|V_{|\mathcal{T}|}|$=n;
- at most c meta-paths connecting ncRNAs and diseases are considered;
- every node is described by m attributes.

In the worst case, a meta-path involves all (i.e., $|\mathcal{R}|$) the types of edges. Therefore, assuming that nodes are indexed according to an order-preserving data structure, the identification of the sequences for all the c considered meta-paths requires $O(c \cdot |\mathcal{R}| \cdot 2n)$ steps. On the other hand, the computation of the similarities among sequences that start with a given ncRNA and end with a given disease, when there is no sequence that directly connects them, is $O(c \cdot n^2 \cdot m \cdot |\mathcal{R}|)$, where $m \cdot |\mathcal{R}|$ corresponds to the cost of computing a single similarity. This means that the complexity of the first phase is: $O(c \cdot (|\mathcal{R}| \cdot 2n + n^2 \cdot m \cdot |\mathcal{R}|))$, which, since the factor $n^2 \cdot m \cdot |\mathcal{R}|$ dominates over $|\mathcal{R}| \cdot 2n$ and since c is a small constant, can be summarized as:

$$O(n^2 \cdot m \cdot |\mathcal{R}|) \quad (1)$$

As for the identification of the hierarchy of clusters, we assume that each ncRNA (resp., disease) is linked, on average, to $\gamma \ll n$ diseases (resp., ncRNAs), according to the considered meta-paths. Therefore, we have $O(\gamma^2)$ ncRNA-disease pairs to consider in the analysis. If every cluster contains $O(\gamma^2)$, the pairwise evaluation of the clusters, would require $O(\gamma^4)$ steps. By assuming that the number of clusters halves at each iteration (which happens if each cluster can be merged with another cluster), the number of iterations is $O(log_2(\gamma^2))$. Therefore, the complexity for the construction of the hierarchy of clusters can be approximated to:

$$O(\gamma^4 \cdot log_2(\gamma^2)) \quad (2)$$

The complexity of the final phase for the prediction of new ncRNA-disease relationships depends on the number of all the possible ncRNA-disease pairs, i.e., $O(|V_n| \cdot |V_d|) = O(n^2)$. In particular, each pair will be associated with a degree of certainty according to the cohesiveness of the clusters it belongs to. Assuming to store the cohesiveness of all the clusters during the construction of the hierarchy and by indexing each ncRNA-disease pair according to the clusters

containing it, this phase only requires a scan of all the possible ncRNA-disease pairs, that is:

$$O(n^2) \tag{3}$$

In short, by combining Equations (1), and (2) and (3), we have that the time complexity of the whole method is:

$$O(n^2 \cdot m \cdot |\mathcal{R}| + \gamma^4 \cdot log_2(\gamma^2)) \tag{4}$$

which strongly depends on the value of γ.

If the dataset, according to the meta-paths, has a relatively small number of links (i.e., $\gamma^4 \cdot log_2(\gamma^2) \leq n^2 \cdot m \cdot |\mathcal{R}|$), the time complexity is $O(n^2 \cdot m \cdot |\mathcal{R}|)$. Otherwise, the time complexity is $O(\gamma^4 \cdot log_2(\gamma^2))$.
